# GWAS identifies an *NAT2* acetylator status tag single nucleotide polymorphism to be a major locus for skin fluorescence

**DOI:** 10.1007/s00125-014-3286-9

**Published:** 2014-06-17

**Authors:** Karen M. Eny, Helen L. Lutgers, John Maynard, Barbara E. K. Klein, Kristine E. Lee, Gil Atzmon, Vincent M. Monnier, Jana V. van Vliet-Ostaptchouk, Reindert Graaff, Pim van der Harst, Harold Snieder, Melanie M. van der Klauw, David R. Sell, S. Mohsen Hosseini, Patricia A. Cleary, Barbara H. Braffett, Trevor J. Orchard, Timothy J. Lyons, Kerri Howard, Ronald Klein, Jill P. Crandall, Nir Barzilai, Sofiya Milman, Danny Ben-Avraham, LifeLines Cohort Study Group, DCCT/EDIC Research Group, Bruce H. R. Wolffenbuttel, Andrew D. Paterson

**Affiliations:** 1 Program in Genetics and Genomic Biology, Hospital for Sick Children, 686 Bay Street, Room 12.9830, Toronto, ON M5G 0A4 Canada; 2 Department of Endocrinology, University Medical Center Groningen, University of Groningen, HPC AA31, PO Box 30001, 9700 RB Groningen, the Netherlands; 3 VeraLight, Inc, Albuquerque, NM USA; 4 Department of Ophthalmology and Visual Sciences, University of Wisconsin School of Medicine and Public Health, Madison, WI USA; 5 Department of Medicine, Institute for Aging Research and the Diabetes Research Center, Albert Einstein College of Medicine, Bronx, NY USA; 6 Department of Genetics, Albert Einstein College of Medicine, Bronx, NY USA; 7 Department of Pathology, Case Western Reserve University, Cleveland, OH USA; 8 Department of Biochemistry, Case Western Reserve University, Cleveland, OH USA; 9 Department of Cardiology, University Medical Center Groningen, University of Groningen, Groningen, the Netherlands; 10 Unit of Genetic Epidemiology and Bioinformatics, Department of Epidemiology, University Medical Center Groningen, University of Groningen, Groningen, the Netherlands; 11 Biostatistics Center, George Washington University, Rockville, MD USA; 12 Department of Epidemiology, University of Pittsburgh, Pittsburgh, PA USA; 13 Centre for Experimental Medicine, Institute of Clinical Science, Queen’s University of Belfast, Belfast, UK; 14 Dalla Lana School of Public Health, University of Toronto, Toronto, ON Canada

**Keywords:** Acetylation, Genome-wide association study, *NAT2*, Skin autofluorescence, Skin fluorescence, Skin intrinsic fluorescence

## Abstract

**Aims/hypothesis:**

Skin fluorescence (SF) is a non-invasive marker of AGEs and is associated with the long-term complications of diabetes. SF increases with age and is also greater among individuals with diabetes. A familial correlation of SF suggests that genetics may play a role. We therefore performed parallel genome-wide association studies of SF in two cohorts.

**Methods:**

Cohort 1 included 1,082 participants, 35–67 years of age with type 1 diabetes. Cohort 2 included 8,721 participants without diabetes, aged 18–90 years.

**Results:**

rs1495741 was significantly associated with SF in Cohort 1 (*p* < 6 × 10^−10^), which is known to tag the *NAT2* acetylator phenotype. The fast acetylator genotype was associated with lower SF, explaining up to 15% of the variance. In Cohort 2, the top signal associated with SF (*p* = 8.3 × 10^−42^) was rs4921914, also in *NAT2*, 440 bases upstream of rs1495741 (linkage disequilibrium *r* ^2^ = 1.0 for rs4921914 with rs1495741). We replicated these results in two additional cohorts, one with and one without type 1 diabetes. Finally, to understand which compounds are contributing to the *NAT2*–SF signal, we examined 11 compounds assayed from skin biopsies (*n* = 198): the fast acetylator genotype was associated with lower levels of the AGEs hydroimidazolones of glyoxal (*p* = 0.017).

**Conclusions/interpretation:**

We identified a robust association between *NAT2* and SF in people with and without diabetes. Our findings provide proof of principle that genetic variation contributes to interindividual SF and that *NAT2* acetylation status plays a major role.

**Electronic supplementary material:**

The online version of this article (doi:10.1007/s00125-014-3286-9) contains peer-reviewed but unedited supplementary material, which is available to authorised users.

## Introduction

AGEs accrue in the body during ageing, and their formation and accumulation are significantly accelerated in diabetes [1]. AGE formation is a multistep process in which the non-enzymatic glycation of proteins is followed by the formation of stable adducts and cross-links, leading to structural and functional tissue impairment [2]. Among people without diabetes, additive genetic effects explain 74% of the population variance of serum-determined AGEs, but the loci are unknown [3].

Skin collagen undergoes glycation and glycoxidation [1]. Given that skin collagen has a half-life of 10–15 years [4], skin AGEs capture decades-long glycaemia [5, 6]. AGEs in skin biopsies predict microvascular complications of type 1 diabetes, independent of HbA_1c_ level [5–7]. However, skin biopsies are impracticable for large studies.

The measurement of skin fluorescence (SF) using optical spectroscopy, corrected for pigmentation, offers a non-invasive measurement of AGEs [8–10]. SF reflects glycaemia in type 1 and type 2 diabetes [8, 11–13] and can screen for abnormal glucose tolerance [14]. SF has been associated with macro- and microvascular complications in type 1 diabetes, independent of long-term HbA_1c_ level [15–18]. In addition, SF is significantly associated with mortality from CHD in diabetes, independent of other risk factors [11, 19].

When adjusted for age and HbA_1c_ level, SF is correlated (*r* = 0.43, *p* = 0.01) between siblings discordant for type 1 diabetes, suggesting genetic contributions [20]. A twin study has shown that lens fluorescence is significantly heritable [21]. However, the specific genetic determinants of SF are unknown. Given that SF is greater in people with than without diabetes [8], we performed genome-wide association studies (GWASs) in two parallel discovery cohorts to identify loci associated with SF in participants with type 1 diabetes and without diabetes.

## Methods

### Design

The first cohort included 1,082 participants with type 1 diabetes from the Diabetes Control and Complications Trial/Epidemiology of Diabetes Interventions and Complications (DCCT/EDIC) study (Table 1, electronic supplementary material [ESM] Table 1) [12]. The second cohort consisted of 8,721 participants without diabetes from the LifeLines Cohort Study (Table 2, ESM Table 2) [22]. Two additional cohorts contributed to replication of the results (ESM Tables 3–5). The first of these cohorts included participants with type 1 diabetes from the Wisconsin Epidemiologic Study of Diabetic Retinopathy (WESDR, *n* = 202) [23] and the second included an older cohort of participants from the LonGenity study (*n* = 515) [24], consisting primarily of participants without diabetes. All individuals were of European descent. These studies were approved by the institutional review boards of all the participating institutions, and informed consent was obtained from all the participants.

**Table 1:** DCCT/EDIC participant characteristics for those with GWAS and SIF measures (*n* = 1,082) taken at the time of SIF assessment shown separately by the original DCCT randomised treatment groups

Variable	Former INT	Former CON
	*n* = 555	*n* = 527
Demographic characteristics		
Male sex	294 (53%)	285 (54%)
Age (years)	52.0 ± 7.0	51.0 ± 6.9
Diabetes duration (years)	30.1 ± 4.9	29.5 ± 4.8
Primary cohort assignment^a^	264 (48%)	270 (51%)
Skin tone (AU)	263.9 ± 39.5	259.4 ± 42.9
Clinic latitude (>37° N)^b^	407 (73%)	393 (75%)
Never smoker^c^	335 (60%)	318 (60%)
Former smoker^c^	143 (26%)	140 (27%)
Current smoker^c^	77 (14%)	69 (13%)
Any eGFR <60 ml min^−1^ 1.73 m^−2^ to date (yes)	36 (7%)	41 (8%)
Glycaemic exposure		
DCCT eligibility HbA_1c_		
%	9.1 ± 1.6	8.9 ± 1.6
mmol/mol	76 ± 18	74 ± 18
DCCT mean HbA_1c_		
%	7.2 ± 0.8	9.0 ± 1.2
mmol/mol	55 ± 9	75 ± 13
EDIC mean HbA_1c_		
%	8.0 ± 1.1	7.9 ± 1.0
mmol/mol	64 ± 12	63 ± 11
Time-weighted mean HbA_1c_^d^		
%	8.0 ± 0.9	8.4 ± 0.9
mmol/mol	64 ± 10	68 ± 10
SIF1 (LED 375 nm, *k*_x_ 0.6, *k*_m_ 0.2) (AU)^e^	3.1 ± 0.2	3.1 ± 0.2
SIF12 (LED 435 nm, *k*_x_ 0.4, *k*_m_ 0.9) (AU)^e^	0.94 ± 0.24	0.94 ± 0.25

Data are *n* (%) or mean ± SD

^a^Two cohorts were recruited at the DCCT baseline: a primary cohort (*n* = 726) of participants with a duration of diabetes of 1–5 years, no retinopathy and a urinary AER <40 mg/24 h at baseline; and a secondary cohort (*n* = 715) of participants with a duration of diabetes of 1–15 years, mild to moderate non-proliferative retinopathy and a urinary AER ≤200 mg/24 h at baseline

^b^Clinic latitude was categorised as a binary variable, with clinics located above 37° N latitude designated as northern clinics (*n* = 21) and those below 37° N latitude assigned as southern clinics (*n* = 7)

^c^Smoking status was defined as ‘Never smoker’ (≤100 cigarettes in a participant’s lifetime), ‘Former smoker’ (quit ≥1 year ago) or ‘Current smoker’ (currently smoking or smoking within the last year)

^d^Time-weighted mean HbA_1c_ is calculated by summing (DCCT eligibility HbA_1c_ × duration of diabetes at DCCT baseline), (DCCT mean HbA_1c_ × years of follow-up in DCCT) and (EDIC mean HbA_1c_ × years of follow-up in EDIC) and dividing by the total duration of diabetes

^e^log_*e*_ transformed

CON, conventional group; INT, intensive group

**Table 2:** LifeLines Cohort Study participant characteristics of those with GWAS and SAF measures available (*n* = 9,039)

Variable	Individuals without diabetes (*n* = 8,721)	Individuals with type 2 diabetes (*n* = 318)
Demographic characteristics		
Male sex	3,590 (41%)	168 (53%)
Age (years)	49.0 ± 11	58.9 ± 10.8
BMI (kg/m^2^)	26.4 ± 4.2	30.5 ± 5.4
Current smoker	1,922 (22%)	55 (17%)
Creatinine (μmol/l)	74 ± 14	78 ± 36
eGFR Cockcroft–Gault (ml min^−1^ 1.73 m^−2^)	113 ± 31	118 ± 45
Glycaemic exposure		
Fasting plasma glucose (mmol/l)	5.0 ± 0.5	7.9 ± 2.3
HbA_1c_		
%	5.5 ± 0.3	6.8 ± 1.1
mmol/mol	37 ± 3.3	52 ± 12
Lipids		
Total cholesterol (mmol/l)	5.1 ± 1.0	4.7 ± 1.2
LDL-cholesterol (mmol/l)	3.3 ± 0.9	2.9 ± 1.0
HDL-cholesterol (mmol/l)	1.44 ± 0.38	1.23 ± 0.32
Triacylglycerol (mmol/l)	1.05 (0.76–1.48)	1.41 (1.04–2.15)
SAF (AU)	2.04 ± 0.44	2.45 ± 0.59

Data are shown as *n* (%), mean ± SD, or median (interquartile range)

### Discovery cohort 1: DCCT/EDIC

#### Participants

At a point 16–17 years after the close-out of the DCCT [25], SF was assessed in 1,082 active participants who had GWAS data [12].

#### Measurement of SF

Skin intrinsic fluorescence (SIF), corrected for factors that affect light scattering and absorption, was measured from the underside of the left forearm using the SCOUT DS SF spectrometer (VeraLight, Inc., Albuquerque, NM, USA) [12, 15]. SIF excited with a light-emitting diode (LED) centred at 375 nm, and emission detected over 435–655 nm (with the reflectance adjusted by the dimensionless exCitation and emission exponents, *k*_x_ = 0.6, *k*_m_ = 0.2, respectively), referred to as SIF1, was the primary outcome [15, 18]. In secondary analyses, we examined SIFs measured using excitation LEDs centred at 405 nm, 416 nm, 435 nm and 456 nm (SIF2–SIF15; ESM Table 6).

### Discovery cohort 2: LifeLines

#### Participants

The LifeLines observational follow-up study includes a random sample of 165,000 inhabitants of three northern provinces of the Netherlands, who have been recruited since 2006 [22]. For this analysis, we included participants 18–90 years of age who had had both GWAS and AGE Reader (DiagnOptics Technologies BV, Groningen, the Netherlands) measurement of skin autofluorescence (SAF) collected from 2006 to 2012. For the GWAS, we excluded participants who were known to have type 1 (*n* = 12) or type 2 diabetes and/or had a fasting blood plasma glucose level >7.0 mmol/l (*n* = 318), leaving 8,721 individuals. We also examined participants with type 2 diabetes (Table 2), either previously known (*n* = 216) or newly diagnosed by fasting blood plasma glucose level (>7.0 mmol/l, *n* = 102) in secondary analyses.

#### Measurement of SF

SAF was measured with the AGE Reader [8, 13]. Similar to SIF1, the excitation light source used a peak at 370 nm. The spectrometer measures emitted and reflected light over 420–600 nm and 300–420 nm, respectively. AGE Reader software (v2.3) calculates SAF from the ratio between the emitted and the reflected light, multiplied by 100 (expressed as arbitrary units [AU]), taking skin colour into account [26]. For each SAF value, measurements were carried out at three different sites of the same forearm, and the mean was used for the analysis.

### Skin collagen ancillary substudy

Skin biopsies were obtained on a subset of DCCT participants (*n* = 216) at close-out, and 11 AGEs and collagen cross-linking variables were determined [5, 6].

### Genotyping

The Illumina 1 M beadchip assay (Illumina, San Diego, CA, USA), which underwent extensive quality control [27], was used in the DCCT/EDIC cohort for genome-wide genotyping. Quality control checks were applied to ensure that there were no sample mix-ups, and participants were excluded if they were determined to be admixed using population genetic approaches [28]. A total of 841,342 autosomal single nucleotide polymorphisms (SNPs) with a minor allele frequency (MAF) >1% were subsequently analysed. In addition, 1,609,583 (INFO ≥ 0.30) imputed autosomal SNPs using release 22 Phase II Centre d'Etude du Polymorphisme (Utah residents with northern and western European ancestry) (CEU) HapMap data (IMPUTE v2; https://mathgen.stats.ox.ac.uk/impute/impute.html) were used for analysis.

In the LifeLines cohort, Illumina CytoSNP 12v2 was used for genome-wide genotyping. Genotyped SNPs with an MAF <0.01, a call rate ≤95% and a Hardy–Weinberg equilibrium (HWE) *p* value <10^−4^ were excluded. A total of 837,184 genotyped or imputed SNPs (allelic *R* ^2^ ≥ 0.80) using HapMap (release 24) CEU (Beagle, v3.1.0) were used for GWAS. Sample relatedness was assessed by participant self-report and population genetic approaches, and the sample with the highest genotyping quality was included for first-degree relatives. Samples were further excluded due to discrepancy in sex, an average heterozygosity >4SD from the mean and non-European ancestry assessed using identical-by-state analysis and Eigenstrat (http://genetics.med.harvard.edu/reich/Reich_Lab/Software.html) [29].

### Statistical analysis

#### GWAS

The primary outcome variable for GWAS in the DCCT/EDIC group was log_e_ SIF1. Since genetic variants may be associated with SF through effects on factors associated with SF, such as smoking or HbA_1c_ level, we first performed a univariate GWAS of SIF1 using genotyped SNPs by linear regression (Model 1 [M1]). To increase the power to detect loci associated with SIF1 by explaining additional variance in the trait, a second GWAS adjusted for age, sex, smoking status, skin tone, clinic latitude and ever having had an estimated GFR (eGFR) <60 ml/min/1.73 m^2^ (Model 2 [M2]). Finally, a third GWAS was conducted adjusting for the same covariates in M2 with the addition of HbA_1c_ measured as follows: at the DCCT eligibility screening, as the mean during the DCCT and as the mean during the EDIC study (M3). DCCT/EDIC Model 3 [M3] was also analysed using imputed SNPs as dosages. To determine whether there were any independent signals, a GWAS conditioning on the top SNP observed from the GWAS was conducted using M1.

In the LifeLines cohort, the primary outcome variable for GWAS was SAF. We first performed an analysis without covariates (M1). Next, we corrected for age, sex and smoking status (M2). Finally, we added BMI, fasting plasma glucose level, HbA_1c_ level, Cockcroft–Gault eGFR and principal components (PCs) to M2 (M3). An additive model was used in all models. A *p*-value <5 × 10^−8^ was required for genome-wide significance in each discovery cohort.

Using METAL (v2011-03-25; www.sph.umich.edu/csg/abecasis/Metal/download/), we also ran a meta-GWAS for M3 using genotyped or imputed SNPs from the DCCT/EDIC and LifeLines studies using weights proportional to √*n* on *z* scores [30].

#### Characterisation of rs1495741 in the DCCT/EDIC

Further analyses were conducted using DCCT/EDIC M1 including adjustment for PCs, and testing for deviation from an additive genetic model by including a heterozygous indicator to a model with rs1495741 coded additively. We also examined the association of rs1495741 with SIF2–SIF15. The heterogeneity of rs1495741 effects was assessed by including rs1495741–covariate interactions. rs1495741 was tested for association with glycaemia measured by HbA_1c_ level during the DCCT and EDIC studies, and with capillary glucose during the DCCT, using linear regression adjusted for DCCT treatment group. The association with the presence of diabetes complications was tested using logistic regression adjusted for covariates in M2 and M3 (each with and without adjustment for any eGFR <60 ml/min/1.73 m^2^). Logistic regression was also used to test rs1495741 for an association with the risk of hypoglycaemia during the DCCT, comparing participants with one or more episodes of hypoglycaemia requiring assistance with those without, and similarly comparing participants with hypoglycaemia resulting in coma with those without. Linear mixed models were used to determine the association with lipids measured annually during the DCCT.

#### Skin biopsy ancillary substudy in the DCCT cohort

Multiple linear regression adjusting for age, duration of diabetes and randomised treatment group in the DCCT was used to determine whether rs1495741 was associated with AGEs measured from skin biopsies [5, 6].

#### Characterisation of rs4921914 in the LifeLines cohort

rs4921914 was tested for an association with HbA_1c_ using linear regression adjusted for age, sex, smoking status, BMI, Cockcroft–Gault eGFR, fasting plasma glucose level and PCs. To test the association with fasting plasma glucose, the same analysis was used, but was adjusted for HbA_1c_ instead of fasting plasma glucose level. The association of rs4921914 with lipid variables was adjusted for all the above covariates with lipid levels adjusted for statin use [31].

#### Testing rs4921914 among type 2 diabetes in the LifeLines cohort

Linear regression was used to test whether rs4921914 was associated with SAF in 318 participants with type 2 diabetes in the LifeLines study. To determine whether the association for rs4921914 differed between participants with type 2 diabetes (*n* = 318) and those without diabetes (*n* = 8,721), we added an rs4921914–diabetes interaction.

PLINK (v1.07) (http://pngu.mgh.harvard.edu/~purcell/plink/) [32] was used for GWASs of genotyped SNPs in DCCT/EDIC, PLINK (v1.90alpha) in LifeLines, R (v2.15.2; www.r-project.org/) for imputed SNPs and for the generation of plots and calculation of genomic control lambda (GenABEL; www.genabel.org/). SAS (v9.2; Cary, NC, USA) and STATA (v.11; College Station, TX, USA) were used for all analyses at the top SNP in the DCCT/EDIC and LifeLines cohorts, respectively.

## Results

### Discovery GWAS 1: DCCT/EDIC

**Fig. 1:**
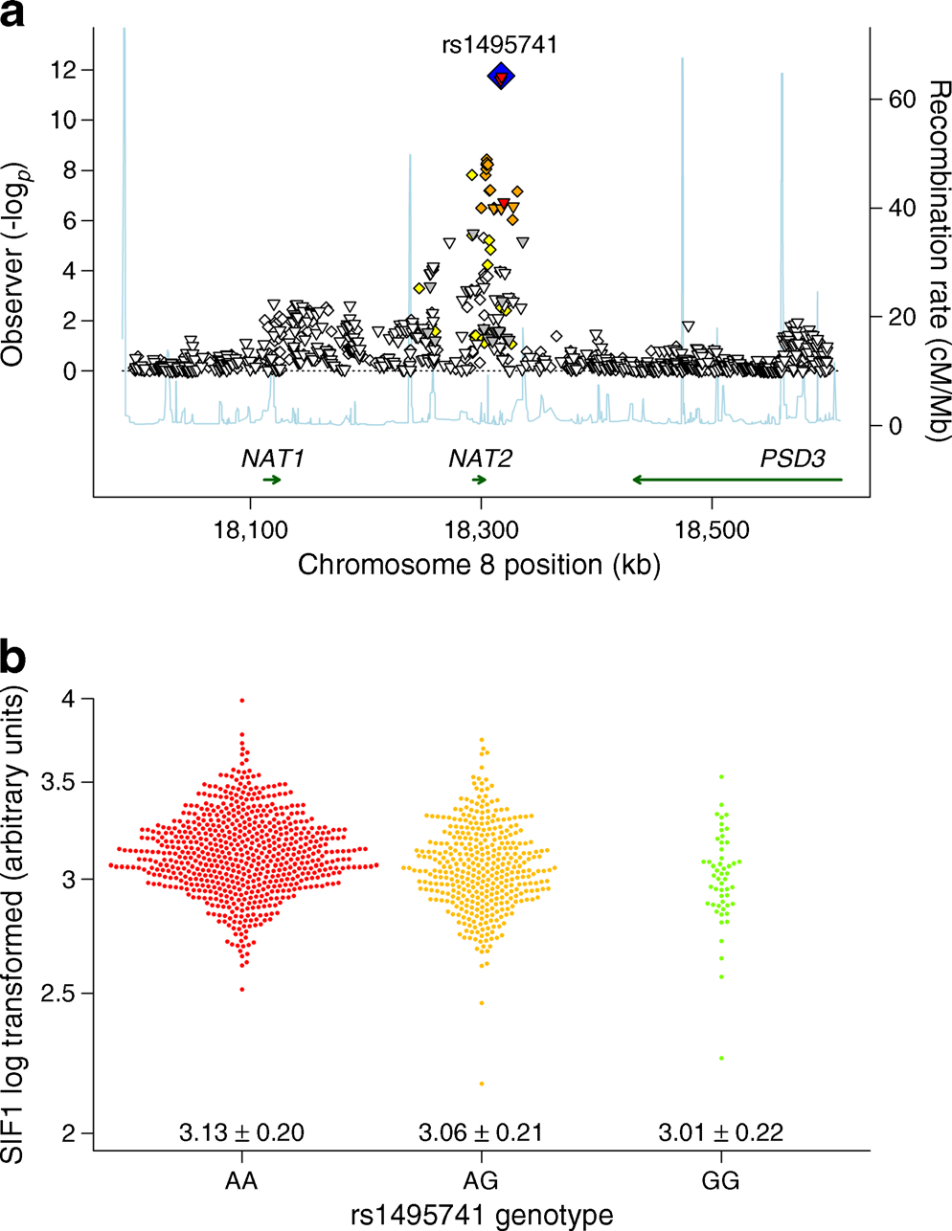
(**a**) Regional plot of a 300 kb region surrounding rs1495741 (*p* = 1.7 × 10^−12^) showing genotyped and imputed SNPs plotted with their (–log_10_) *p* values from the DCCT/EDIC cohort (M3) on the left *y*-axis and their genomic position (NCBI Build 35; www.ncbi.nlm.nih.gov/mapview/stats/BuildStats.cgi?taxid=9606&build=3) on the *x*-axis. Gene annotations (Genome Browser; http://genome.ucsc.edu/) are shown above the *x*-axis. Estimated recombination rates (HapMap II release 22; http://hapmap.ncbi.nlm.nih.gov/) are plotted on the right *y*-axis. For genotyped SNPs, the LD values shown were calculated based on pairwise *r* ^2^ values for rs1495741 from the DCCT/EDIC cohort, and for imputed SNPs are based on *r* ^2^ values from HapMap phase II (Nov08, release 24, on NCBI B36 assembly, dbSNP b126). The blue diamond indicates rs1495741 and the SNPs are coloured based on their LD with it (red, *r* ^2^ ≥ 0.8; orange, 0.5 ≤ *r* ^2^ < 0.8; yellow, 0.2 ≤ *r* ^2^ < 0.5; white, *r* ^2^ < 0.2); (www.broadinstitute.org/diabetes/scandinavs/figures.html). (**b**) Beeswarm plot showing level of unadjusted log_*e*_ SIF1 for each participant in the DCCT/EDIC cohort according to their rs1495741 genotype, with the mean ± SD shown above the *x*-axis

The GWAS of SIF1 from the DCCT/EDIC cohort identified a significant association with rs1495741 (*p* = 6.1 × 10^−10^; Table 3), 14 kb downstream of *NAT2* (Fig. 1a, ESM Fig. 1a). rs1495741 is associated with the in vitro measured *NAT2* acetylator phenotype and tags haplotypes that define the acetylator status with 99% sensitivity and 95% specificity in white individuals [33–35]. Examination of the rs1495741 allele intensity cluster plot showed a clear separation of genotypes (ESM Fig. 2, G-allele MAF = 0.22, chromosome 8p22, build 36 position 18,317,161), with no significant deviation from HWE (*p* = 0.75). The association with SIF1 was not materially different after adjusting for the first three PCs (*p* for M1 = 5.5 × 10^−10^). Upon adjusting for covariates in M2 and M3, no additional genome-wide significant loci were identified (ESM Fig. 1b, c, ESM Fig. 3, Table 3, GWAS results available from dbGaP: ftp://ftp.ncbi.nlm.nih.gov/dbgap/studies/phs000086/analyses/). Results from an analysis conditional on rs1495741 did not identify any independent signals that were significant genome-wide (data not shown). SIF1 was lower with each copy of the fast acetylator G-allele (Fig. 1b) and there was no deviation from an additive model (*p* = 0.69). Results from M1 show that rs1495741 explains 3.5% of the variance in SIF1, and explains an additional 3% of the variance when added to the covariates in M3, which explains 33% of the variance in SIF1 [12].

**Table 3:** Associations of rs1495741 with SIF, glycaemia, complications of type 1 diabetes, AGEs and lipids in the DCCT/EDIC cohort

Variable	*n* (AA/AG/GG)	β±SE/OR (95% CI)	*p* value
SIF			
SIF1 M1 (AU)	1,081 (649/380/52)	−0.065 ± 0.010	6.1 × 10^−10^
SIF1 M2 (AU)	1,081 (649/380/52)	−0.059 ± 0.009	9.3 × 10^−12^
SIF1 M3 (AU)	1,081 (649/380/52)	−0.060 ± 0.008	1.7 × 10^−12^
SIF12 M3 (AU)	1,081 (649/380/52)	−0.16 ± 0.01	2.9 × 10^−49^
Glycaemia			
HbA_1c_ updated mean (%)^a,b^	1,081 (649/380/52)	−0.0003 ± 0.006	0.95
Mean 7-point capillary blood glucose profile (mmol/l)^a^	1,303 (782/458/63)	0.010 ± 0.009	0.23
T1DM complications^c^			
Moderate non-proliferative DR or worse	1,081 (649/380/52)	1.20 (0.97, 1.49)	0.09
Sustained AER >30 mg/24 h	1,081 (649/380/52)	0.89 (0.64,1.24)	0.47
Presence of confirmed clinical neuropathy	1,018 (610/359/49)	1.02 (0.80, 1.30)	0.90
Presence of cardiac autonomic neuropathy	1,046 (629/365/52)	0.90 (0.71, 1.14)	0.37
CAC >0 Agatston units	978 (582/348/48)	1.07 (0.83, 1.39)	0.60
CAC >200 Agatston units	978 (582/348/48)	0.81 (0.50, 1.30)	0.38
Hypoglycaemia			
Hypoglycaemia requiring assistance	1,303 (782/458/63)	0.97 (0.81, 1.17)	0.78
Hypoglycaemia resulting in coma or seizure	1,303 (782/458/63)	0.89 (0.72, 1.09)	0.25
Skin biopsy AGEs			
Pepsin soluble collagen (% solubility)^a^	198 (132/61/5)	0.08 ± 0.06	0.13
Acid soluble collagen (% solubility)^a^	198 (132/61/5)	0.01 ± 0.07	0.87
*N* ^ε^-Carboxymethyl-lysine (pmol/mg collagen)	196 (131/60/5)	−8.84 ± 17.4	0.61
Fluorescence (AU)^a^	198 (132/61/5)	−0.03 ± 0.03	0.27
Furosine (pmol/mg collagen)^a^	195 (129/61/5)	0.001 ± 0.03	0.97
Pentosidine (pmol/mg collagen)^a^	194 (130/59/5)	−0.03 ± 0.03	0.31
Carboxyethyl-lysine (pmol/mg)^a^	196 (130/61/5)	0.17 ± 0.09	0.059
Glucosepane (nmol/mg)^a^	198 (132/61/5)	0.01 ± 0.09	0.73
Hydroimidazolones of methylglyoxal (nmol/mg)^a^	198 (132/61/5)	0.05 ± 0.007	0.43
Fructose-lysine (nmol/mg)^a^	198 (132/61/5)	0.02 ± 0.04	0.60
G-H1 (pmol/mg)^b^	197 (131/61/5)	−0.62 ± 0.26	0.017
Lipids^d^			
Total cholesterol (mmol/l)^a^	1,303 (782/458/63)	0.004 ± 0.008	0.57
LDL-cholesterol (mmol/l)^e^	1,303 (782/458/63)	0.01 ± 0.009	0.27
HDL-cholesterol (mmol/l)^a^	1,303 (782/458/63)	−0.03 ± 0.01	0.008
Triacylglycerol (mmol/l)^a^	1,303 (782/458/63)	0.04 ± 0.02	0.01

Data shown are β±SE from linear regression or mixed linear models for continuous outcomes, or OR (95% CI) from logistic regression for the SNP effect, with each copy of the G-allele coded additively

^a^Log_*e*_ transformed

^b^Time-weighted mean HbA_1c_ is calculated by summing (DCCT eligibility HbA_1c_ × duration of diabetes at DCCT baseline), (DCCT mean HbA_1c_ × years of follow-up in DCCT) and (EDIC mean HbA_1c_ × years of follow-up in EDIC) and dividing by the total duration of diabetes

^c^Results shown are from logistic regression analyses adjusted for M2 covariates

^d^The linear mixed model analyses of lipids were adjusted for visit, visit^2^, DCCT treatment group, DCCT baseline indicator and DCCT treatment group × DCCT baseline indicator interaction Random effects were included for the intercept and visit, visit^2^

^e^Square root transformed

CAC, coronary artery calcium; DR, diabetic retinopathy; T1DM, type 1 diabetes

We also examined the association for rs1495741 with SIF2–SIF15 and observed the strongest relationship for SIF12 (*p* for M3 = 2.9 × 10^−49^), explaining 15.0% of the variance (Table 3, ESM Table 6). Importantly, rs1495741 was still significantly associated with SIF1 (*p* = 4.04 × 10^−19^) after adjusting for SIF12, suggesting that the effect on SIF1 is independent of the effect on SIF12.

### Discovery GWAS 2: LifeLines

The top SAF association signals observed in the LifeLines cohort were 47 SNPs that were also in the *NAT2* region (M3; *p* < 5 × 10^−8^) (Table 4, ESM Figs 4 and 5). The top signal (LifeLines M3, *p* = 1.0 × 10^−60^, *R* ^2^ = 2.1%) was rs4921914, 440 bases upstream of rs1495741 (ESM Fig. 6). According to HapMap phase 2 CEU (release 22), rs4921914 is in perfect linkage disequilibrium (LD; *r* ^2^ = 1.0) with rs1495741 (C- and G-alleles, respectively). rs4921914 was imputed with allelic *R* ^2^ = 0.81 (MAF = 0.19 for the C-allele) and showed no deviation from HWE (*p* = 0.36). The association was in the same direction as observed in the DCCT/EDIC group.

**Table 4:** Associations of rs4921914 with SAF, glycaemia and lipids in the LifeLines Cohort Study

Variable	*n*	β±SE	*p* value
SAF			
M1 (AU)	8,721	−0.1149 ± 0.0084	8.3 × 10^−42^
M2 (AU)	8,721	−0.1154 ± 0.0069	8.2 × 10^−62^
M3 (AU)	8,675	−0.1148 ± 0.0069	1.0 × 10^−60^
Glycaemia			
Fasting plasma glucose (mmol/l)	8,698	−0.009 ± 0.009	0.36
HbA_1c_ (%)	8,689	−0.005 ± 0.006	0.36
Lipids			
Total cholesterol (mmol/l)	8,706	−0.011 ± 0.019	0.56
LDL-cholesterol (mmol/l)	8,706	−0.004 ± 0.017	0.80
HDL-cholesterol (mmol/l)	8,705	−0.00014 ± 0.007	0.98
Triacylglycerol (mmol/l)^a^	8,706	0.019 ± 0.009	0.06

Data shown are β±SE from linear regression for the SNP effect with each copy of the C-allele coded additively

^a^Log_*e*_ transformed

We did not identify any additional genome-wide significant loci after meta-GWAS (ESM Figs 7–8).

### Heterogeneity of rs1495741 effect on SF

In the DCCT/EDIC cohort, rs1495741 was not significantly associated with any of the covariates in M2 (*p* > 0.05, data not shown). There was no heterogeneity of rs1495741 on SIF1 (ESM Table 7), with the exception of the updated weighted mean HbA_1c_ levels for the DCCT/EDIC, which approached nominal significance (*p* for the SNP*HbA_1c_ interaction = 0.058). When analysing the effect of rs1495741 separately by the median updated weighted mean HbA_1c_ level for the DCCT/EDIC (8.07%), rs1495741 was stronger among participants with HbA_1c_ levels greater than median (β±SE = −0.075 ± 0.01, *p* = 4.7 × 10^−7^) compared with those below the median (β±SE = −0.054 ± 0.01, *p* = 1.2 × 10^−4^). In the LifeLines group, no significant SNP × HbA_1c_ interaction was observed among the non-diabetic participants (*p* = 0.28). However, there was a significant heterogeneity of rs4921914 by age (*p* = 0.03).

### Biochemical measures and complications of type 1 diabetes

In the DCCT/EDIC cohort, rs1495741 was not associated with updated weighted mean HbA_1c_ up to the time of SIF measurement (*p* = 0.95; Table 3). In participants without diabetes in the LifeLines cohort, rs4921914 was also not associated with HbA_1c_ (*p* = 0.36) or fasting plasma glucose (*p* = 0.36) level (Table 4). Furthermore, rs1495741 was not associated with the mean of 7-point capillary blood glucose profiles measured during the DCCT (*p* = 0.23). In the DCCT/EDIC cohort, rs1495741 was not associated with any microvascular complications or markers of macrovascular complications in models adjusted for M2 and/or M3 covariates (either with or without adjusting for any eGFR<60 ml/min/1.73 m^2^), or with hypoglycaemia (Table 3).

Since rs1495741 has been associated with lipids [36], we examined its association with repeated measures of lipids during the DCCT. rs1495741 was nominally associated with triacylglycerol (*p* = 0.01) and HDL-cholesterol (*p* = 0.008) values (Table 3). The effect of rs1495741 on SIF1 was unchanged after adjusting for time-weighted mean triacylglycerol (*p* = 1.8 × 10^−12^) or HDL-cholesterol (*p* = 9.1 × 10^−13^) on M3. In the LifeLines cohort, no association could be demonstrated (Table 4), although the power to detect an effect on triacylglycerol and total cholesterol [36] was 61% and 31%, respectively.

### Skin biopsy ancillary substudy in the DCCT cohort

Of the 11 AGEs and collagen cross-linking variables measured in the skin biopsies, rs1495741 was associated only with hydroimidazolones of glyoxal (G-H1; *p* = 0.017; Table 3), in the same direction as the association with SF.

### SAF among individuals with type 2 diabetes in the LifeLines cohort

rs4921914 was significantly associated (β±SE = −0.20 ± 0.06, *p* = 5.2 × 10^−4^) with SAF in participants with type 2 diabetes and explained 3.4% of the variance in SAF. The difference in the SNP effect between the participants with and without type 2 diabetes was *p* = 0.07, with a stronger association observed in those with type 2 diabetes.

### Replication in the WESDR and LonGenity cohorts

In a second cohort of type 1 diabetes participants, rs1495741 (G-allele MAF = 25%, HWE *p* = 0.51) was associated with SIF1 (β±SE = −0.06 ± 0.02, *p* for the multivariate model = 0.002, *r* ^2^ for the univariate model = 5.3%), in the same direction of effect as in the discovery cohorts (Table 5). The effect of rs1495741 on SIF1 did not differ according to smoking status (*p* = 0.54). rs1495741 was also associated with SIF14 (β±SE = 0.10 ± 0.02, *p* for the multivariate model = 6.0 × 10^−5^, *r* ^2^ for the univariate model = 9.2%). Finally, rs1495741 was not associated with repeated measures of HbA_1c_ in linear mixed models (*p* = 0.53), or time to mild (*p* = 0.28) or severe diabetic retinopathy (*p* = 0.18).

**Table 5:** Associations of rs1495741 with SIF1, SIF14 and other measures in the WESDR and LonGenity cohorts

Variable	*n*	β±SE	*p* value
WESDR			
SIF1 (AU)^a,b^	200	−0.06 ± 0.02	0.002
SIF14 (AU)^a,c^	202	−0.10 ± 0.02	6.0 × 10^−5^
HbA_1c_ (%)^c^	601	0.05 ± 0.08	0.53
Mild DR^d^	603	0.08 ± 0.08	0.28
Severe DR^d^	603	0.13 ± 0.10	0.18
LonGenity			
SIF1 (AU)^e,f^	515	−0.01 ± 0.007	0.09
SIF14 (AU)^e,f^	515	−0.02 ± 0.007	0.0004

Data shown are β±SE from linear regression for the SNP effect with each copy of the G-allele coded additively

^a^Log_*e*_ transformed

^b^Two outliers (one at each tail) were observed for SIF1 in the WESDR cohort and excluded from the analysis, leaving 200 participants for analysis of SIF1. The SNP was examined for association with SIF1 and SIF14 adjusting for age, sex, smoking status, skin tone, eGFR <60 ml min^−1^ 1.73 m^−2^ and HbA_1c_ concurrent with the SIF measure

^c^Associations of the SNP with repeated measures of HbA_1c_ were analysed using linear mixed models adjusted for time and random intercept

^d^Complementary log–log models for interval-censored survival times were used to examine associations with mild diabetic retinopathy (DR), adjusted for age at baseline, sex, duration of diabetes at baseline, time-dependent updated mean HbA_1c_ and time-dependent BMI, and severe DR (adjusted for the same variables except for time-dependent BMI).

^e^log_10_ transformed

^f^Association for the SNP with SIF1 and SIF14 was examined adjusting for age, sex, skin tone, smoking status, eGFR <60 ml/min/1.73 m^2^ and presence of diabetes

DR, diabetic retinopathy

rs1495741 (G-allele MAF = 18%, HWE *p* = 0.92) was not significantly associated with SIF1 (β±SE = −0.01 ± 0.007, *p* = 0.09; Table 5) in LonGenity, an older cohort (mean ± SD age 75 ± 6 years) of participants predominantly without diabetes (89%). However, consistent with the DCCT/EDIC and WESDR cohorts, rs1495741 was associated with SIF14 (β±SE = −0.02 ± 0.007, *p* = 0.0004) in the same direction.

## Discussion

Most GWASs of quantitative traits use blood-based measures, for which levels may vary widely within a person over time due to clearance predominantly by the kidneys and liver. SF, on the other hand, provides a measure of long-term tissue damage associated with ageing [8, 9] as well as with decades-long blood glucose levels in people with diabetes [12], given the long half-life of skin collagen [4]. Using a GWAS, we identified rs1495741 located 14 kb downstream of *NAT2* to be associated with SF in individuals with type 1 diabetes, and the same signal (rs4921914, *r* ^2^ = 1.0) was observed in a separate discovery cohort of individuals without diabetes. We further replicated the association for SF in individuals with type 1 and 2 diabetes. In addition, the signal observed for rs1495741 with SIF14 was also replicated in the WESDR and LonGenity populations. In the DCCT/EDIC cohort, rs1495741 explains 3.5% of the variance in SIF1 and appears to be tagging *NAT2*, with no signals observed in the neighbouring *NAT1*. In all four studies, each copy of the fast acetylator allele was associated with less SF.

The association of *NAT2* with SF was robust across different populations and different measures of SF both within and across studies. First, we observed the association in individuals both with and without type 2 diabetes in the LifeLines group, as well as in those with type 1 diabetes in the DCCT/EDIC and WESDR cohorts. There was suggestion that a stronger effect of *NAT2* on SF might exist in individuals with type 2 diabetes than in those without diabetes. Similarly, the effect of *NAT2* on SIF was stronger in participants who had higher HbA_1c_ levels in comparison to those with lower HbA_1c_ levels in the DCCT/EDIC population. However, no significant SNP*HbA_1c_ interaction was observed among non-diabetic participants in the LifeLines group. Second, *NAT2* was strongly associated with SF excited across the range of 375–456 nm in the DCCT/EDIC group, with the strongest effect observed for excitation at 435 nm (SIF12). Finally, *NAT2* was the top signal observed in both discovery GWAS cohorts, despite using different SF devices. Indeed, both the SCOUT DS and the AGE Reader, used in the LifeLines study, have been shown to correlate with skin biopsy-determined AGEs such as pentosidine [8, 9].

Although SF has been associated with HbA_1c_ level in people with diabetes [8, 11, 12], rs1495741 was not associated with glycaemia in people either with or without diabetes in our cohorts. However, in the Meta-Analyses of Glucose and Insulin-related traits Consortium (MAGIC; http://www.magicinvestigators.org), a large meta-GWAS of more than 46,000 non-diabetic adults of European descent, the G-allele of rs1495741 was associated with higher HbA_1c_ (*p* = 0.003) [37] and fasting plasma glucose (*p* = 0.03) values [38]. Both associations were in the opposite direction to the effect of *NAT2* on SF (ESM Table 8). Thus, a small effect for rs1495741 on glycaemic traits in non-diabetic individuals may exist and may be observed only in very large analyses.

Despite the association of SF with the complications of type 1 diabetes [15–18, 39], rs1495741 was not associated with coronary artery calcium, nephropathy, neuropathy or retinopathy in the DCCT/EDIC group, or with time from baseline to either mild or severe retinopathy in the WESDR cohort. The non-significant association with these outcomes is due to the low power to detect small effects, since we have good power for detecting an OR >1.50 for a sustained AER >30 mg/24 h with α = 0.05 [15]. In line with this, the rs1495741 G-allele has previously been associated (OR 1.06, *p* = 2 × 10^−5^) with an increased risk of coronary artery disease (CAD) [36].

Of the 11 AGEs and collagen cross-linking variables assayed, rs1495741 was nominally associated with G-H1 in the same direction as with SF. G-H1 is a hydroimidazolone derived from arginine residues modified by glyoxal, a potent glycating agent [40]. G-H1 itself is not fluorescent, but its reactive AGE precursor, glyoxal, can participate in the formation of fluorescent structures such as vesperlysine A, which is detected at 370 nm/440 nm excitation/emission [41]. In the DCCT, G-H1 was positively associated with age, but not with duration of diabetes or with recent or long-term HbA_1c_ levels [6]. Finally, on its own, G-H1 was not significantly associated with risk of any of the microvascular complications in the DCCT [6]. Although G-H1 was not correlated with SIF1 measured 15 years later (ESM Table 9), this may be due to its half-life of 2–6 weeks [40]. Replication of the association of *NAT2* with G-H1 is needed in individuals with diabetes, as is a determination of the association in those without diabetes.

We used SIF1 for our GWAS in the DCCT/EDIC cohort based on previous observations that SIF1 had the strongest association with complications [15, 18] and was closest to the excitation wavelength used in the LifeLines study. However, SF measures can be obtained from a wide range of excitation/emission spectra, which reflect different fluorophores [8, 42]. For example, SF measured with 370 nm/440 nm excitation/emission has been shown to capture AGEs in general and has been shown to correlate with pentosidine levels determined by skin biopsy [42]. The less studied 440 nm/520 nm excitation/emission fluorescence, which closely corresponds with SIF12, correlated with pentosidine as well as with *N* ^ε^-carboxymethyl-lysine, carboxyethyl-lysine and methionine sulphoxide [42]. However, *NAT2* was not associated with these AGEs in the DCCT skin biopsy substudy. Thus, our observation of a stronger association for *NAT2* with SIF12 in the DCCT/EDIC cohort may also reflect other AGEs that are possibly associated with elastin–collagen cross-links detected in response to excitation at higher wavelengths [42]. SIF12, however, also coincides with non-AGE fluorophores including flavin adenine dinucleotide and oxidised flavin mononucleotide [10], as well as phospholipids, and therefore the *NAT2* signal for SIF12 may occur through non-AGE mechanisms [43]. Follow-up studies are needed to determine the compounds responsible for the association between *NAT2* and SF.

*N*-Acetyltransferase 2 (NAT2) is known to metabolise drugs and carcinogens, but no known endogenous substrates have been identified [44]. A previous GWAS identified an association of rs1495741 with bladder cancer risk, with significant evidence for a gene-by-smoking interaction [45]. However, we observed no such heterogeneity of effect for rs1495741 on SIF1 by smoking status. *NAT2* has also been identified in several other GWASs, including those involving plasma [46] and urine metabolites [47] as well as lipids [36]. In both the previous meta-GWAS [36] and the DCCT, the fast acetylator G-allele was associated with higher triacylglycerol levels. Unlike the previous study of primarily individuals without diabetes [36], rs1495741 was associated with lower HDL-cholesterol values among fast acetylators in the DCCT cohort (Table 3, ESM Table 10). Importantly, the effect of rs1495741 on SIF1 was unchanged after adding time-weighted mean triacylglycerol or HDL-cholesterol to M3, arguing that the genetic effect on SF is not mediated by triacylglycerol or HDL-cholesterol. The mechanism(s) linking the fast *NAT2* acetylator genotype with both elevated triacylglycerol levels and CAD is unknown [36]. However, the opposing direction of effect of rs1495741 on SF in comparison to triacylglycerol, CAD and possibly glycaemic traits [37, 38] suggests that multiple underlying pathways may exist.

The LifeLines study used the AGE Reader to measure SAF with a peak excitation of 370 nm, compared with SIF1 measured in the DCCT/EDIC study using the SCOUT DS with an excitation peak at 375 nm, and different corrections were made for skin pigmentation. Despite this limitation, the *NAT2* region was robustly the top signal associated with both SIF1 in the DCCT/EDIC and SAF in the LifeLines cohort. Accordingly, in the DCCT/EDIC cohort, a correlation of *r*_pearson_ = 0.69 (*p* 10^−15^) was observed for SIF1 with a proxy for SAF (LED 375 nm, *k*_x_ = 1.0, *k*_m_ = 0.0), showing that SIF and SAF are comparable measurements. *NAT2* was not significantly associated with SIF1 in the LonGenity study (*p* = 0.09), possibly due to the age of this cohort, given that collagen production is altered with older age [48]. However, it was associated with SIF14. Although NAT2 activity and protein levels have not been detected in normal human dermal fibroblasts [49], the metabolism of substrates by NAT2 in the liver or colon may contribute to the differences in SF between slow and fast acetylators [44, 50]. Finally, since not all AGEs fluoresce, a GWAS of SF may not identify loci specific to the production or detoxification of non-fluorescent AGEs, unless they are highly correlated with a fluorophore in the skin.

In conclusion, using a GWAS, our study demonstrates that genetic variation contributes to SF, and that *NAT2* is a major locus observed across four independent studies. Although *NAT2* was not significantly associated with the risk of complications of type 1 diabetes, larger studies are needed. *NAT2* may improve the screening properties of SF in predicting the risk of complications or impaired glucose tolerance [14–18]. The robust association observed for *NAT2* with SF in multiple cohorts provides proof of principle that genetic variation contributes to the variance in SF.

## Electronic supplementary material

Below is the link to the electronic supplementary material.

ESM Study writing groups (PDF 27 kb)

ESM Table 1 (PDF 179 kb)

ESM Table 2 (PDF 167 kb)

ESM Table 3 (PDF 174 kb)

ESM Table 4 (PDF 102 kb)

ESM Table 5 (PDF 87 kb)

ESM Table 6 (PDF 219 kb)

ESM Table 7 (PDF 210 kb)

ESM Table 8 (PDF 100 kb)

ESM Table 9 (PDF 155 kb)

ESM Table 10 (PDF 96.7 kb)

ESM Fig. 1 (PDF 384 kb)

ESM Fig. 2 (PDF 26 kb)

ESM Fig. 3 (PDF 258 kb)

ESM Fig. 4 (PDF 785 kb)

ESM Fig. 5 (PDF 175 kb)

ESM Fig. 6 (PDF 120 kb)

ESM Fig. 7 (PDF 861 kb)

ESM Fig. 8 (PDF 284 kb)

## Abbreviations

AU: Arbitrary units
CAD: Coronary artery disease
EDIC: Epidemiology of Diabetes Interventions and Complications
eGFR: Estimated GFR
G-H1: Hydroimidazolones of glyoxal
GWAS: Genome-wide association study
HWE: Hardy–Weinberg equilibrium
LD: Linkage disequilibrium
LED: Light-emitting diode
M1: Model 1
M2: Model 2
M3: Model 3
MAF: Minor allele frequency
NAT2: *N*-Acetyltransferase 2
PC: Principal component
SAF: Skin autofluorescence
SF: Skin fluorescence
SIF: Skin intrinsic fluorescence
SNP: Single nucleotide polymorphism
UKPDS: UK Prospective Diabetes Study
WESDR: Wisconsin Epidemiologic Study of Diabetic Retinopathy
