# Supplementary material for: GWAS identifies an NAT2 acetylator status tag single nucleotide polymorphism to be a major locus for skin fluorescence
Source: Diabetologia. 2014 Jun 17;57(8):1623–34. doi: 10.1007/s00125-014-3286-9 (PMC4079945; doi:10.1007/s00125-014-3286-9)
Supplement: Supplementary file 1 — (PDF 27 kb) [file 125_2014_3286_MOESM1_ESM.pdf]

ELECTRONIC SUPPLEMENTARY MATERIAL

STUDY WRITING GROUPS:

LifeLines Cohort Study Group Author:

Behrooz Z Alizadeh (1), Rudolf A de Boer (2), H Marike Boezen (1), Marcel Bruinenberg (3), Lude Franke (4), Pim van der Harst (2), Hans L Hillege (1,2), Melanie M van der Klauw (5), Gerjan Navis (6), Johan Ormel (7), Dirkje S Postma (8), Judith GM Rosmalen (7), Joris P Slaets (9), Harold Snieder (1), Ronald P Stolk (1), Bruce HR Wolffenbuttel (5), Cisca Wijmenga (4)

(1) Department of Epidemiology, University of Groningen, University Medical Center Groningen, The Netherlands

(2) Department of Cardiology, University of Groningen, University Medical Center Groningen, The Netherlands

(3) LifeLines Cohort Study, University of Groningen, University Medical Center Groningen, The Netherlands

(4) Department of Genetics, University of Groningen, University Medical Center Groningen, The Netherlands

(5) Department of Endocrinology, University of Groningen, University Medical Center Groningen, The Netherlands

(6) Department of Internal Medicine, Division of Nephrology, University of Groningen, University Medical Center Groningen, The Netherlands

(7) Interdisciplinary Center of Psychopathology of Emotion Regulation (ICPE), Department of Psychiatry, University of Groningen, University Medical Center Groningen, The Netherlands

(8) Department of Pulmonology, University of Groningen, University Medical Center Groningen, The Netherlands

(9) University Center for Geriatric Medicine, University of Groningen, University Medical Center Groningen, The Netherlands

DCCT/EDIC Research Group:

A complete list of participants in the DCCT/EDIC research group can be found in New England Journal of Medicine, 2011;365:2366-2376 (PMC3270008).
