# Supplementary material for: GWAS identifies an NAT2 acetylator status tag single nucleotide polymorphism to be a major locus for skin fluorescence
Source: Diabetologia. 2014 Jun 17;57(8):1623–34. doi: 10.1007/s00125-014-3286-9 (PMC4079945; doi:10.1007/s00125-014-3286-9)
Supplement: Supplementary file 2 — (PDF 179 kb) [file 125_2014_3286_MOESM2_ESM.pdf]

ESM Table 1: Clinical measures and type 1 diabetes complications measured in DCCT/EDIC.

Measure	Details
**eGFR**	Serum creatinine was measured annually in DCCT/EDIC and was used to calculate estimated glomerular filtration rate (eGFR) from the Chronic Kidney Disease Epidemiology Collaboration equation[1]. Any eGFR<60 ml/min/1.73m^2 from DCCT baseline to the date of the SIF measurement was used as a binary variable[2].
**HbA$_{1c}$**	HbA$_{1c}$ was measured at the eligibility visit, every three months during DCCT and annually during EDIC[3,4.]
**Lipids**	During DCCT, lipids were collected annually and analyzed as previously described[5].
**Glucose**	Seven time-point blood capillary glucose profiles were obtained quarterly with the overall mean calculated for all measures taken within a day and across all time points during DCCT[6].
**Skin tone**	Skin tone was measured non-invasively from the SCOUT DS SF spectrometer (VeraLight, Inc., Albuquerque, NM)[2,7].
**Retinopathy**	Retinopathy was measured by standardized seven-field fundus photography biannually during DCCT. During EDIC approximately one quarter of the cohort was assessed each year, and the entire cohort was examined at years 4 and 10. Presence of retinopathy at the time of the participant's most recent clinical assessment prior to SIF measures as previously described[7] was used as a binary variable.

**Nephropathy**	Albumin excretion rate (AER) was measured annually during the DCCT and on alternate years during the EDIC study using a timed 4-h urine collection and expressed per 24 hours. Presence of nephropathy at the time of the participant’s most recent clinical assessment prior to SIF measures as previously described[7] was used as a binary variable.
**Neuropathy**	Two measures of neuropathy were used, cardiac autonomic neuropathy (CAN) and confirmed clinical neuropathy (CCN). The presence or absence of CAN as previously described[7] was assessed by measuring sinus arrhythmia[8] in EDIC ears 16/17. Presence or absence of CCN as previously described[7] was measured by nerve conduction tests done in EDIC years 13/14.
**Coronary artery calcium (CAC)**	CAC measured by either multi-slice or electron beam computed tomography at EDIC year 12 was used[9]. Two threshold levels were used as binary outcomes, one as presence or absence of CAC >0 and the second as presence or absence of CAC>200 Agatston units[7].
**Hypoglycaemia**	Hypoglycaemia requiring assistance or hypoglycaemia resulting in coma or seizure was collected during DCCT as previously described[10].
**Skin collagen parameters**	Eleven advanced glycation end products and collagen crosslinking parameters from skin biopsies were collected at DCCT close-out on a subset of participants and were assayed at two time points (n=216)[11,12].

[1] Levey AS, Stevens LA, Schmid CH, et al. (2009) A new equation to estimate glomerular filtration rate. Ann Intern Med 150: 604-612
[2] Cleary PA, Braffett BH, Orchard T, et al. (2013) Clinical and technical factors associated with skin intrinsic fluorescence in subjects with type 1 diabetes from the Diabetes Control and Complications Trial/Epidemiology of Diabetes Interventions and Complications Study. Diabetes Technol Ther 15: 466-474
[3] (1999) Epidemiology of Diabetes Interventions and Complications (EDIC) Research Group. Design, implementation, and preliminary results of a long-term follow-up of the Diabetes Control and Complications Trial cohort. Diabetes Care 22: 99-111
[4] (1986) The Diabetes Control and Complications Trial (DCCT). Design and methodologic considerations for the feasibility phase. The DCCT Research Group. Diabetes 35: 530-545
[5] (1995) Effect of intensive diabetes management on macrovascular events and risk factors in the Diabetes Control and Complications Trial. Am J Cardiol 75: 894-903
[6] (1993) The Diabetes Control and Complications Trial Research Group. The effect of intensive treatment of diabetes on the development and progression of long-term complications in insulin-dependent diabetes mellitus. . N Engl J Med 329: 977-986
[7] Orchard TJ, Lyons TJ, Cleary PA, et al. (2013) The association of skin-intrinsic fluorescence with type 1 diabetes complications in the DCCT/EDIC Study. Diabetes Care
[8] Pop-Busui R, Low PA, Waberski BH, et al. (2009) Effects of prior intensive insulin therapy on cardiac autonomic nervous system function in type 1 diabetes mellitus: the Diabetes Control and Complications Trial/Epidemiology of Diabetes Interventions and Complications study (DCCT/EDIC). Circulation 119: 2886-2893
[9] Cleary PA, Orchard TJ, Genuth S, et al. (2006) The effect of intensive glycemic treatment on coronary artery calcification in type 1 diabetic participants of the Diabetes Control and Complications Trial/Epidemiology of Diabetes Interventions and Complications (DCCT/EDIC) Study. Diabetes 55: 3556-3565
[10] (1997) Hypoglycemia in the Diabetes Control and Complications Trial. The Diabetes Control and Complications Trial Research Group. Diabetes 46: 271-286
[11] Monnier VM, Bautista O, Kenny D, et al. (1999) Skin collagen glycation, glycoxidation, and crosslinking are lower in subjects with long-term intensive versus conventional therapy of type 1 diabetes: relevance of glycated collagen products versus HbA1c as markers of diabetic complications. DCCT Skin Collagen Ancillary Study Group, Diabetes Control and Complications Trial. Diabetes 48: 870-880
[12] Monnier VM, Sell DR, Strauch C, et al. (2013) The association between skin collagen glucosepane and past progression of microvascular and neuropathic complications in type 1 diabetes. J Diabetes Complications 27: 141-149
