# Supplementary material for: GWAS identifies an NAT2 acetylator status tag single nucleotide polymorphism to be a major locus for skin fluorescence
Source: Diabetologia. 2014 Jun 17;57(8):1623–34. doi: 10.1007/s00125-014-3286-9 (PMC4079945; doi:10.1007/s00125-014-3286-9)
Supplement: Supplementary file 3 — (PDF 167 kb) [file 125_2014_3286_MOESM3_ESM.pdf]

ESM Table 2: Clinical measures assessed in LifeLines.[a]

Measure	Details
**Lipids**	Total cholesterol was measured with an enzymatic colorimetric method, HDL-cholesterol with a colorimetric method, and triacylglycerol with a colorimetric UV method, on a Roche Modular P chemistry analyzer (Roche, Basel, Switzerland).
**HbA1c**	A fasting whole blood sample (EDTA-anticoagulated) was collected and analysed using a turbidimetric inhibition immunoassay on a Cobas Integra 800 CTS analyser (Roche Diagnostics Nederland BV, Almere, the Netherlands). This method has been standardized against the reference method of the International Federation of Clinical Chemistry and Laboratory Medicine. Between-batch imprecision (coefficient of variation) was 2.1% for a mean HbA_{1c} of 5.5%, and 1.9% for a mean HbA_{1c} of 10.6%[1].
**Fasting plasma glucose**	Measured from fasting fresh venous plasma using the Roche glucose assay (hexokinase/glucose-6-phosphate dehydrogenase enzymatic reactions) on the Modular P analyzer (Roche Diagnostics, Burgdorf, Switzerland).
**Renal function**	Serum creatinine was measured enzymatically on the same equipment. The Cockcroft-Gault (CG) equation was used to estimate renal function, with the following formula: 1.23 × (140 – age)/serum creatinine) × weight (× 0.85 for women).

[a]Blood sampling was performed in the fasting state, between 8 and 10 am. The samples were then transported in temperature-controlled conditions (at room temperature or at 4C depending on the sample requirements) to the LifeLines laboratory facility. Measurements were carried out on fresh samples.

[1] Jansen H, Stolk RP, Nolte IM, Kema IP, Wolffenbuttel BH, Snieder H (2013) Determinants of HbA1c in nondiabetic Dutch adults: genetic loci and clinical and lifestyle parameters, and their interactions in the Lifelines Cohort Study. J Intern Med 273: 283-293
