# Supplementary material for: GWAS identifies an NAT2 acetylator status tag single nucleotide polymorphism to be a major locus for skin fluorescence
Source: Diabetologia. 2014 Jun 17;57(8):1623–34. doi: 10.1007/s00125-014-3286-9 (PMC4079945; doi:10.1007/s00125-014-3286-9)
Supplement: Supplementary file 4 — (PDF 174 kb) [file 125_2014_3286_MOESM4_ESM.pdf]

ESM Table 3: Clinical measures and details for Wisconsin Epidemiologic Study of Diabetic Retinopathy (WESDR).

	**Details**
**Inclusion criteria**	All 'younger onset' participants diagnosed <30 yrs and on insulin (i.e. type 1 diabetes) of 10,135 type 1 and type 2 diabetes patients who received primary care in an 11-county area in southern Wisconsin from 1979-80, were eligible. 996/1,210 participated in the baseline examination (1980–2).
**HbA1c**	Glycosylated hemoglobin A1 levels were measured at baseline, 4, 10 and 14 year follow-ups from capillary blood samples and glycosylated hemoglobin A1c at the 20 and 25 year follow-ups from venous blood (Quick Step Fast Hemoglobin Test System. Akron, OH: Isolab) as previously described[1]. The WESDR glycosylated hemoglobin A1 microcolumn results compare with the Diabetes Control and Complications Trial (DCCT) glycosylated hemoglobin A1c results as follows: DCCT = 0.003 + 0.935 (WESDR).
**Retinopathy**	Stereoscopic color fundus photography of the seven standard fields were performed at every visit, except for the 20 year follow-up visit. Retinopathy severity was graded according to the Early Treatment Diabetic Retinopathy Study (ETDRS) scale of diabetic retinopathy severity. Two endpoints for diabetic retinopathy development were defined as follows: an ETDRS level of at least 31 in the worst eye defined presence of mild diabetic retinopathy, and an

	ETDRS score above 53 in the worst eye or a history of panretinal photocoagulation therapy defined presence of severe diabetic retinopathy.
**SIF**	SIF was measured during the recent 30 year visit currently in progress, using the SCOUT DS skin fluorescence spectrometer (VeraLight, Inc., Albuquerque, NM), as previously described[2,3].
**GWAS**	DNA was collected at the second visit (1984-6) in approximately half of the individuals, and again at 20 and 25 yrs. GWAS genotyping using the Illumina Human Omni-Quad BeadChip was completed on 665 WESDR type 1 diabetes participants as previously described[4]. Two hundred and two participants had both GWAS data and SIF measurements available.

[1] Klein R, Knudtson MD, Lee KE, Gangnon R, Klein BE (2009) The Wisconsin Epidemiologic Study of Diabetic Retinopathy XXIII: the twenty-five-year incidence of macular edema in persons with type 1 diabetes. Ophthalmology 116: 497-503

[2] Cleary PA, Braffett BH, Orchard T, et al. (2013) Clinical and technical factors associated with skin intrinsic fluorescence in subjects with type 1 diabetes from the Diabetes Control and Complications Trial/Epidemiology of Diabetes Interventions and Complications Study. Diabetes Technol Ther 15: 466-474

[3] Orchard TJ, Lyons TJ, Cleary PA, et al. (2013) The association of skin-intrinsic fluorescence with type 1 diabetes complications in the DCCT/EDIC Study. Diabetes Care

[4] Verhoeven VJ, Hysi PG, Wojciechowski R, et al. (2013) Genome-wide meta-analyses of multiancestry cohorts identify multiple new susceptibility loci for refractive error and myopia. Nat Genet 45: 314-318
