# Supplementary material for: GWAS identifies an NAT2 acetylator status tag single nucleotide polymorphism to be a major locus for skin fluorescence
Source: Diabetologia. 2014 Jun 17;57(8):1623–34. doi: 10.1007/s00125-014-3286-9 (PMC4079945; doi:10.1007/s00125-014-3286-9)
Supplement: Supplementary file 5 — (PDF 102 kb) [file 125_2014_3286_MOESM5_ESM.pdf]

ESM Table 4: Clinical measures and details for LonGenity.[a]

**Inclusion Criteria**	Participants aged 65 years or older and of Ashkenazi Jewish descent (all 4 grandparents) are eligible. Among eligible participants, two cohorts of participants are recruited. The first consisting of offspring of parents with exceptional longevity (OPEL), where at least one parent lived in relative good health to age 95 years or older. The second cohort consists of offspring of parents with usual survival (OPUS), in which neither parent lived to age 95 years. The two groups were not deliberately matched for age and sex, although they are similar. 900 participants have been recruited since 2007, and is currently ongoing.
**Exclusion Criteria**	Participants with established dementia were excluded from participation.
**SIF**	SIF was measured at the baseline or wave 1 exam using the SCOUT DS skin fluorescence spectrometer (VeraLight, Inc., Albuquerque, NM), as previously described[1,2].
**GWAS**	Genotyping was performed at the Center for Inherited Disease Research (CIDR) using the HumanOmniExpress (Illumina, San Diego, CA, USA) SNP genome panel. Illumina BeadStudio software (San Diego, CA, USA) was used to evaluate all genotypes using the quantitative GenCall score. The overall genotype call rate was 99.21%.<br>A thorough QC analysis was completed including: Identity by descent (IBD) analysis to determine cryptic relatedness, SNP quality (missing call rate, mendelian errors, Hardy-Weinberg Equilibrium, minor allele

	frequency), batch effects (Median missing call rate, allelic frequency differences, and number of mis-identified samples), sample quality (missing call rate over SNPs, allelic imbalance and heterozygosity). No relatedness and no batch effect was observed, SNP quality was high with low Mendelian errors (Mendelian Consistency Rate of 99.75%), Hardy-Weinberg Equilibrium (average p=0.54), low average missing call rate of 0.33% and low concordance rate (homozygote/heterozygote concordance) of 99.73%. In total, 515 participants had both GWAS data and SIF measurements available.

[a]The LonGenity study is a longitudinal study aimed at ascertaining genetic and biochemical markers for exceptional longevity that may aid in the prevention of frailty, cognitive decline and cardiovascular events.

[1] Cleary PA, Braffett BH, Orchard T, et al. (2013) Clinical and technical factors associated with skin intrinsic fluorescence in subjects with type 1 diabetes from the Diabetes Control and Complications Trial/Epidemiology of Diabetes Interventions and Complications Study. Diabetes Technol Ther 15: 466-474

[2] Orchard TJ, Lyons TJ, Cleary PA, et al. (2013) The association of skin-intrinsic fluorescence with type 1 diabetes complications in the DCCT/EDIC Study. Diabetes Care
