# Supplementary material for: GWAS identifies an NAT2 acetylator status tag single nucleotide polymorphism to be a major locus for skin fluorescence
Source: Diabetologia. 2014 Jun 17;57(8):1623–34. doi: 10.1007/s00125-014-3286-9 (PMC4079945; doi:10.1007/s00125-014-3286-9)
Supplement: Supplementary file 6 — (PDF 87 kb) [file 125_2014_3286_MOESM6_ESM.pdf]

ESM Table 5: Participant characteristics for WESDR (type 1 diabetes) and LonGenity at the time SIF was measured.

	**WESDR (n=202)**	**LonGenity (n=515)**
**Age (years)**	57 ± 8.87	75 ± 6.49
**Male Sex**	107 (53%)	247 (48%)
**Participants with Diabetes**	202 (100%)	59 (11%)
**Duration of diabetes (years)**	42.0 ± 6.88	N/A
**Mean HbA_{1c} (%) (mmol/mol)**	7.8 ±1.2 (62 ± 13.1)	N/A
**Skin tone (arbitrary units)**	262.7 ± 49.3	242±38
**Smoking status**		
**Never smoker**	124 (61%)	N/A
**Former smoker**	62 (31%)	N/A
**Current smoker**	16 (8%)	52%
**eGFR <60 ml/min/1.73m^2 (yes)**	45 (22%)	160 (31%)
**SIF1 raw (arbitrary units)**	25.3 ± 5.34	22.3± 4.57
**SIF1 transformed (arbitrary units)**	3.2 ± 0.21[a]	1.3 ± 0.09[b]
**SIF14 (arbitrary units)**	1.7 ± 0.38	1.4± 0.32
**SIF14 transformed (arbitrary units)**	0.50 ± 0.22[a]	0.15 ± 0.10[b]

Data are n (%) or mean ± SD values. eGFR = estimated GFR; SIF= skin intrinsic fluorescence.

[a]ln transformed.

[b]log_{10} transformed.
