# Supplementary material for: GWAS identifies an NAT2 acetylator status tag single nucleotide polymorphism to be a major locus for skin fluorescence
Source: Diabetologia. 2014 Jun 17;57(8):1623–34. doi: 10.1007/s00125-014-3286-9 (PMC4079945; doi:10.1007/s00125-014-3286-9)
Supplement: Supplementary file 7 — (PDF 219 kb) [file 125_2014_3286_MOESM7_ESM.pdf]

ESM Table 6: Associations of rs1495741 with SIF1 to SIF15 adjusted for model 3 covariates in DCCT/EDIC.

LED level (Emission range)	SIF (k_x, k_m)[a]	Mean ± SD	β ± SE	p-value	Type II squared Semi-partial Correlation
375nm (435-655nm)	SIF1 (Kx0.6 km0.2)	3.10 ± 0.20	-0.060 ± 0.008	1.69E-12	0.029
	SIF2 (Kx0.8 Km0.2)	3.20 ± 0.25	-0.060 ± 0.01	6.75E-10	0.020
	SIF3 (Kx0.4 Km0.7)	2.62 ± 0.19	-0.058 ± 0.008	2.43E-12	0.030
405nm (440-655nm)	SIF4 (Kx0.6 Km0.2)	2.11 ± 0.23	-0.12 ± 0.009	1.35E-35	0.090
	SIF5 (Kx0.8 Km0.2)	2.11 ± 0.23	-0.12 ± 0.0095	9.45E-34	0.089
	SIF6 (Kx0.9 Km0.0)	2.27 ± 0.24	-0.12 ± 0.0097	1.45E-32	0.085
416nm (451-655nm)	SIF7 (Kx0.8 Km0.2)	1.84 ± 0.24	-0.14 ± 0.0099	5.32E-42	0.116
	SIF8 (Kx0.9 Km0.0)	2.00 ± 0.24	-0.14 ± 0.010	4.64E-41	0.113
	SIF9 (Kx0.4 Km0.9)	1.28 ± 0.23	-0.14 ± 0.0098	2.15E-42	0.124
435nm (470-655nm)	SIF10 (Kx0.9 Km0.0)	1.59 ± 0.25	-0.16 ± 0.0104	5.92E-48	0.137
	SIF11 (Kx0.4 Km0.8)	1.02 ± 0.25	-0.16 ± 0.0103	2.77E-49	0.144
	SIF12 (Kx0.4 Km0.9)	0.94 ± 0.24	-0.16 ± 0.0103	2.93E-49	0.146
456nm (491-655nm)	SIF13 (Kx0.9 Km0.0)	0.68 ± 0.24	-0.12 ± 0.0099	6.51E-33	0.090
	SIF14 (Kx0.4 Km0.8)	0.36 ± 0.23	-0.12 ± 0.0097	1.43E-34	0.097
	SIF15 (Kx0.4 Km0.9)	0.28 ± 0.23	-0.12 ± 0.0097	1.57E-34	0.099

Data shown are mean ± SD (N=1082) for each SIF as well as β ± SE from linear regression for the SNP effect with each copy of the G-allele coded additively (N=1081).

LED= light emitting diode; SIF= skin intrinsic fluorescence; kx= excitation correction factor; km= emission correction factor. The squared semi-partial correlation coefficients were computed using Type II sums of squares.

[a]All the SIF variables were ln transformed.
