# Supplementary material for: GWAS identifies an NAT2 acetylator status tag single nucleotide polymorphism to be a major locus for skin fluorescence
Source: Diabetologia. 2014 Jun 17;57(8):1623–34. doi: 10.1007/s00125-014-3286-9 (PMC4079945; doi:10.1007/s00125-014-3286-9)
Supplement: Supplementary file 8 — (PDF 210 kb) [file 125_2014_3286_MOESM8_ESM.pdf]

ESM Table 7: Testing heterogeneity of rs1495741 effect (rs1495741-by-environment interaction) on SIF[a] in DCCT/EDIC.

	**β±SE**	**p-value**
**rs1495741*Age**	0.001 ± 0.001	0.38
**rs1495741*Sex (Men vs Women)**	-0.03 ± 0.02	0.10
**rs1495741*smoker (Never smokers vs Ever and Current smokers)**	0.03164 ± 0.02	0.13
**rs1495741*any eGFR<60 ml/min/1.73m^2**	0.07 ± 0.04	0.08
**rs1495741*Time-weighted mean HbA_{1c}[b]**	-0.02 ± 0.01	0.058
**rs1495741*T1DM duration**	-0.0008 ± 0.002	0.69
**rs1495741*former DCCT treatment group (INT vs CON)**	-0.003 ± 0.02	0.88
**rs1495741*DCCT cohort (primary prevention vs. secondary intervention)**	0.003 ± 0.02	0.87

Data shown are β ± SE and p-value from linear regression testing for the SNP-by-environment interaction.

SIF= skin intrinsic fluorescence; T1DM = type 1 diabetes.

[a]SIF was $\log_e$ transformed.

[b]Time-weighted mean HbA_{1c} is calculated by summing (DCCT eligibility HbA_{1c} x duration of diabetes at DCCT baseline), (DCCT mean HbA_{1c} X years of follow-up in DCCT), and (EDIC mean HbA_{1c} X years of follow-up in EDIC) and dividing by total duration of diabetes.
