# Supplementary material for: GWAS identifies an NAT2 acetylator status tag single nucleotide polymorphism to be a major locus for skin fluorescence
Source: Diabetologia. 2014 Jun 17;57(8):1623–34. doi: 10.1007/s00125-014-3286-9 (PMC4079945; doi:10.1007/s00125-014-3286-9)
Supplement: Supplementary file 9 — (PDF 100 kb) [file 125_2014_3286_MOESM9_ESM.pdf]

**ESM Table 8:** Results from previously published meta-GWASs for the association of rs1495741 (A>G) with: $HbA_{1c}$ [1] and fasting glucose [2]

| Trait | N | Beta Estimate or Odds Ratio (For each copy of the G allele) | StdErr or 95% Confidence Interval | P-value |
|---|---|---|---|---|
| **$HbA_{1c}$ (%)** | 46,368 | 0.0122 | 0.004 | 0.003 |
| **Fasting glucose (mmol/l)** | 58,074 | 0.008 | 0.004 | 0.03 |

N represents sample size for each study. Beta estimate or odds ratio effect sizes are modelled as an additive effect for each copy of the G allele.

**Meta-GWAS results were downloaded from**: www.magicinvestigators.org (August 22, 2013).


[1] Soranzo N, Sanna S, Wheeler E et al. (2010) Common variants at 10 genomic loci influence hemoglobin A(1)(C) levels via glycemic and nonglycemic pathways. Diabetes 59: 3229-3239

[2] Manning AK, Hivert MF, Scott RA et al. (2012) A genome-wide approach accounting for body mass index identifies genetic variants influencing fasting glycemic traits and insulin resistance. Nat Genet 44: 659-669
