# Supplementary material for: GWAS identifies an NAT2 acetylator status tag single nucleotide polymorphism to be a major locus for skin fluorescence
Source: Diabetologia. 2014 Jun 17;57(8):1623–34. doi: 10.1007/s00125-014-3286-9 (PMC4079945; doi:10.1007/s00125-014-3286-9)
Supplement: Supplementary file 10 — (PDF 155 kb) [file 125_2014_3286_MOESM10_ESM.pdf]

ESM Table 9: Spearman correlations for each of the skin biopsy variables and SIF1 measured 16 or 17 years later in DCCT/EDIC.

		Unadjusted		Adjusted	
	n	Spearman r	p-value	Spearman r	p-value
Pepsin Soluble Collagen (% Solubility)	185	-0.28	0.0001	-0.14	0.052
Acid Soluble Collagen (% Solubility)	185	-0.05	0.48	0.04	0.62
N^{ε}-carboxymethyl-lysine (pmoles/mg collagen)	183	0.35	<.0001	0.16	0.03
Fluorescence (arbitrary units)	185	0.31	<.0001	0.12	0.12
Furosine (pmoles/mg collagen)	182	0.23	0.0021	0.29	<.0001
Pentosidine (pmoles/mg collagen)	181	0.35	<.0001	0.08	0.29
Carboxyethyl-lysine (pmol/mg)	184	0.03	0.72	-0.01	0.90
Glucosepane (nmol/mg)	185	0.42	<.0001	0.23	0.002
Hydroimidazolones of methylglyoxal (nmol/mg)	185	0.22	0.003	0.06	0.45
Fructose-lysine (nmol/mg)	185	0.15	0.04	0.20	0.005
Hydroimidazolones of glyoxal (pmol/mg)	184	0.09	0.23	0.03	0.72

The adjusted Spearman correlations used SIF1 and skin biopsy variables each residually adjusted for both age and type 1 diabetes duration at the time of each respective measure.
