# Supplementary material for: GWAS identifies an NAT2 acetylator status tag single nucleotide polymorphism to be a major locus for skin fluorescence
Source: Diabetologia. 2014 Jun 17;57(8):1623–34. doi: 10.1007/s00125-014-3286-9 (PMC4079945; doi:10.1007/s00125-014-3286-9)
Supplement: Supplementary file 11 — (PDF 96.7 kb) [file 125_2014_3286_MOESM11_ESM.pdf]

ESM Table 10: Results from previous meta-GWAS for the association of rs1495741 (A>G) with lipids [1].

**Lipid**	Weight	Effect (For each copy of the G allele)	StdErr	P-value
**Total cholesterol (mg/dL)**	97,148	0.0324	0.0061	2.70×10^{-8}
**LDL-cholesterol (mg/dL)**	92,503	0.0185	0.0063	0.002
**HDL-cholesterol (mg/dL)**	96,908	-0.0027	0.0058	0.93
**Triglycerides (mg/dL)**	93,562	0.0434	0.0057	4.61×10^{-14}

Weight represents the sum of the individual study weights (typically, N). Effect size estimates are shown to correspond to the standard deviation unit increase or decrease for each lipid modelled as an additive effect for each copy of the G allele. StdErr is the standard error for effect size estimates. The p-value shown is the meta-analysis p-value, after genomic control correction.

Meta-GWAS results were downloaded from:

http://www.sph.umich.edu/csg/abecasis/public/lipids2010/ (August 26, 2013).

[1]] Teslovich TM, Musunuru K, Smith AV et al. (2010) Biological, clinical and population relevance of 95 loci for blood lipids. Nature 466: 707-713
