# Supplementary material for: GWAS identifies an NAT2 acetylator status tag single nucleotide polymorphism to be a major locus for skin fluorescence
Source: Diabetologia. 2014 Jun 17;57(8):1623–34. doi: 10.1007/s00125-014-3286-9 (PMC4079945; doi:10.1007/s00125-014-3286-9)
Supplement: Supplementary file 12 — (PDF 384 kb) [file 125_2014_3286_MOESM12_ESM.pdf]

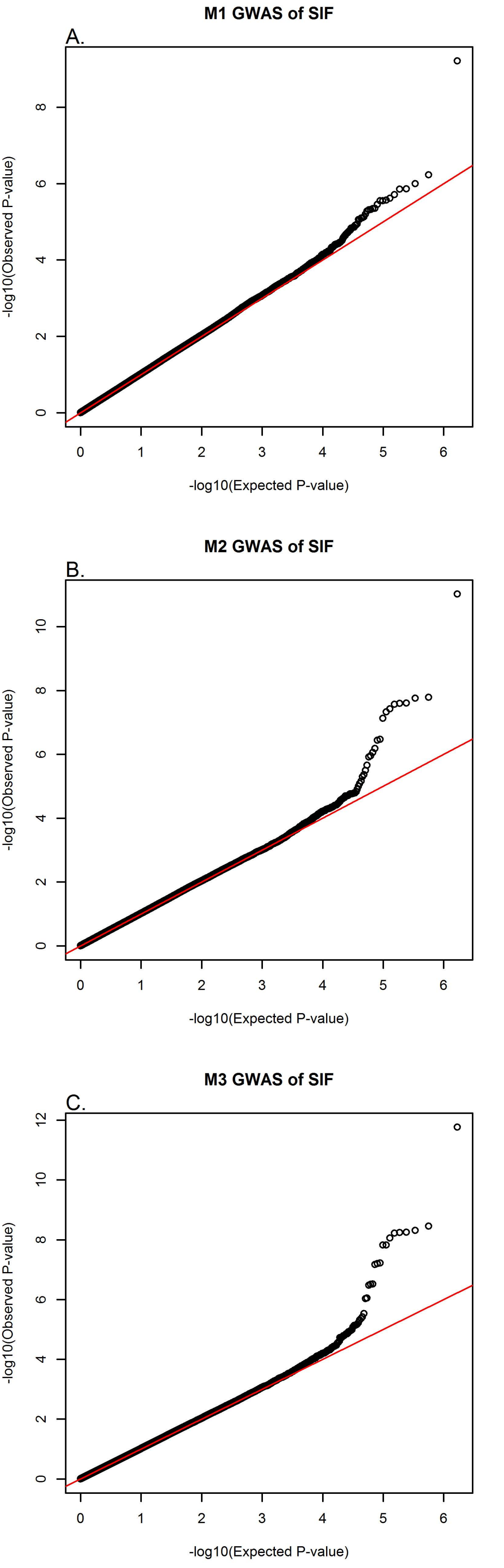
M1 GWAS of SIF
A.
-log10(Observed P-value)
-log10(Expected P-value)
M2 GWAS of SIF
B.
-log10(Observed P-value)
-log10(Expected P-value)
M3 GWAS of SIF
C.
-log10(Observed P-value)
-log10(Expected P-value)

ESM Figure 1: Q-Q plots of the observed versus expected quantiles of –log10(pvalues) from 841,342 genotyped SNP associations with lnSIF1. **A,** M1: Univariate; **B,** M2: adjusted for age, sex, smoking status, skin tone, clinic latitude and any eGFR<60 ml/min/1.73m^2; **C,** M3: adjusted for covariates in M2 + HbA_{1c} at DCCT eligibility, mean HbA_{1c} during DCCT, and mean HbA_{1c} during EDIC. Genomic control lambda for SIF1 were all equal to 1.02 (M1-M3).
