# Supplementary material for: GWAS identifies an NAT2 acetylator status tag single nucleotide polymorphism to be a major locus for skin fluorescence
Source: Diabetologia. 2014 Jun 17;57(8):1623–34. doi: 10.1007/s00125-014-3286-9 (PMC4079945; doi:10.1007/s00125-014-3286-9)
Supplement: Supplementary file 13 — (PDF 26 kb) [file 125_2014_3286_MOESM13_ESM.pdf]

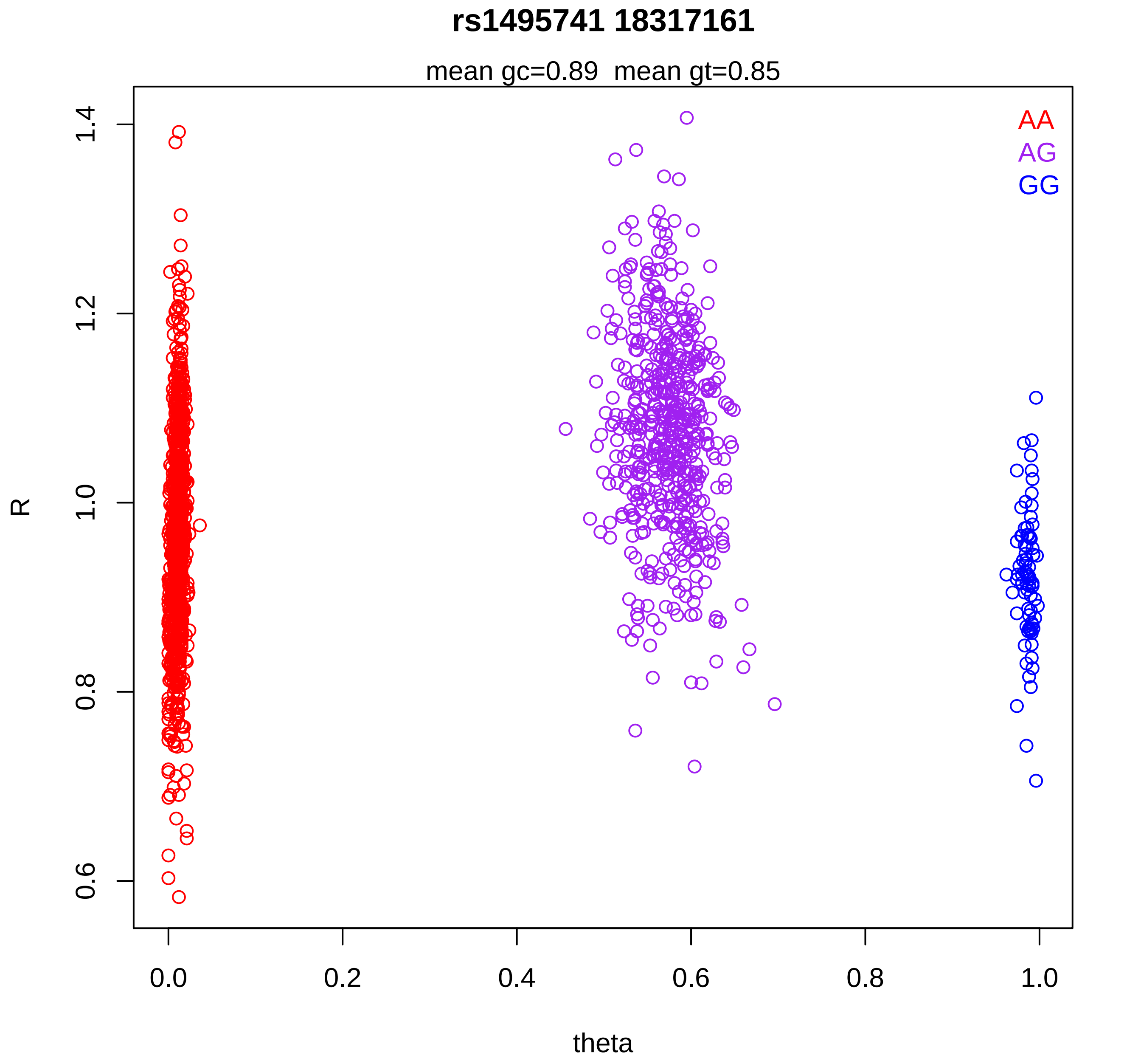

rs1495741 18317161
mean gc=0.89 mean gt=0.85
AA
AG
GG
R
theta
0.6
0.8
1.0
1.2
1.4
0.0
0.2
0.4
0.6
0.8
1.0

ESM Figure 2: The cluster plot for rs1495741 shows polar coordinates, where x=0 represent a pure A-allele signal and x=1 represents a pure G-allele signal and the y-axis represents the distance of the point to the origin (intensity for allele A = intensity for allele G). Red, purple and blue circles represent the AA, AG and GG genotype groups, respectively. A clear separation of rs1495741 genotypes can be seen for participants in DCCT/EDIC. Only one participant had missing genotype information for rs1495741.
