# Supplementary material for: GWAS identifies an NAT2 acetylator status tag single nucleotide polymorphism to be a major locus for skin fluorescence
Source: Diabetologia. 2014 Jun 17;57(8):1623–34. doi: 10.1007/s00125-014-3286-9 (PMC4079945; doi:10.1007/s00125-014-3286-9)
Supplement: Supplementary file 14 — (PDF 258 kb) [file 125_2014_3286_MOESM14_ESM.pdf]

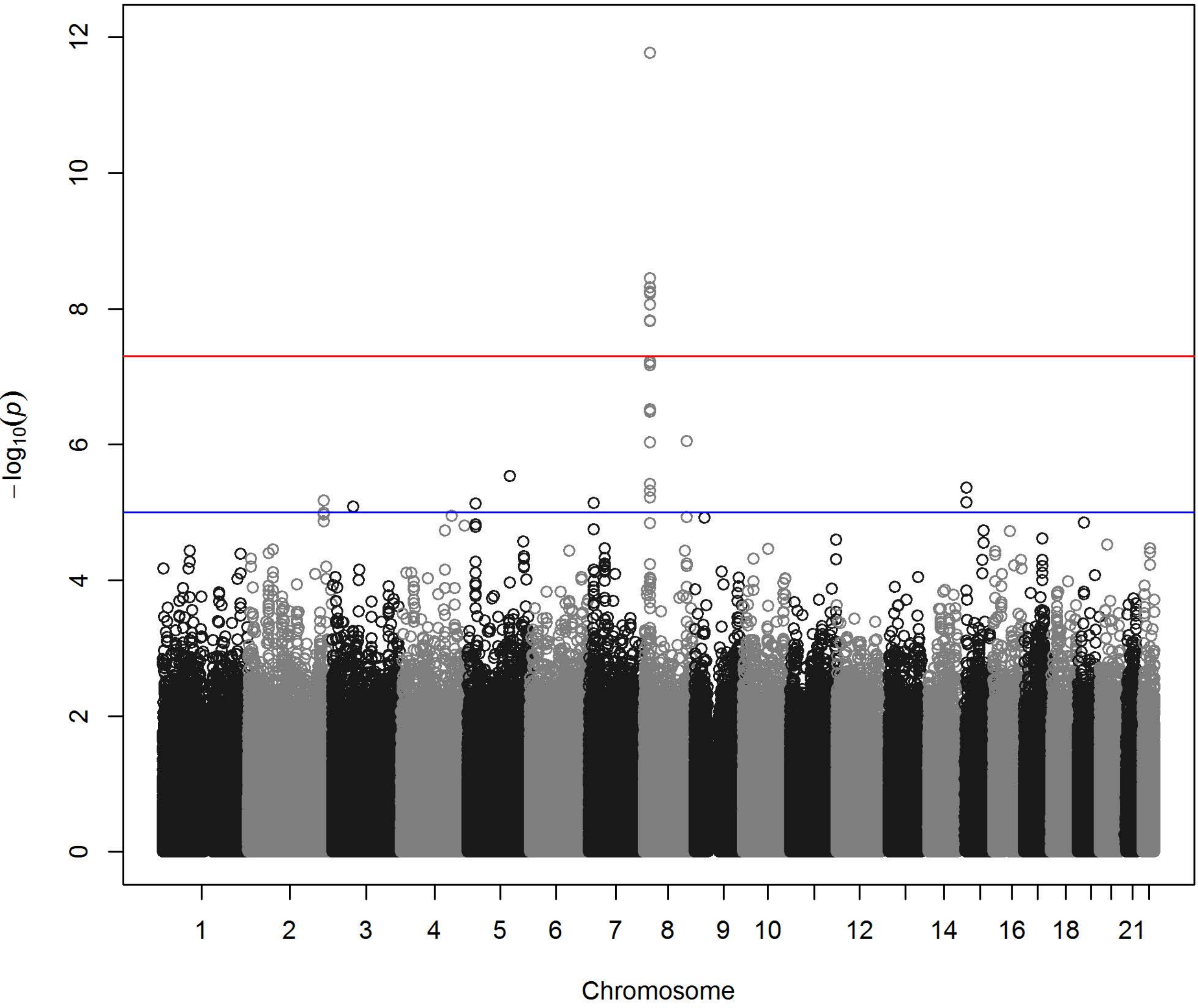

0
2
4
6
8
10
12
−log10(p)
1
2
3
4
5
6
7
8
9
10
12
14
16
18
21
Chromosome

ESM Figure 3: The Manhattan plot for the SIF1 (Model 3) GWAS in DCCT/EDIC shows $-\log_{10} P$ values (*y*-axis) of 841,342 SNPs plotted against their chromosomal positions (*x*-axis). The red horizontal line represents the genome-wide significance threshold, p=5x10-8; the blue line represents p=1x10-5. The Manhattan plot was generated using R code written by Getting Genetics Done (http://gettinggeneticsdone.blogspot.ca/2010/01/gwas-manhattan-plots-and-qq-plots-using.html).
