# Supplementary material for: GWAS identifies an NAT2 acetylator status tag single nucleotide polymorphism to be a major locus for skin fluorescence
Source: Diabetologia. 2014 Jun 17;57(8):1623–34. doi: 10.1007/s00125-014-3286-9 (PMC4079945; doi:10.1007/s00125-014-3286-9)
Supplement: Supplementary file 15 — (PDF 785 kb) [file 125_2014_3286_MOESM15_ESM.pdf]

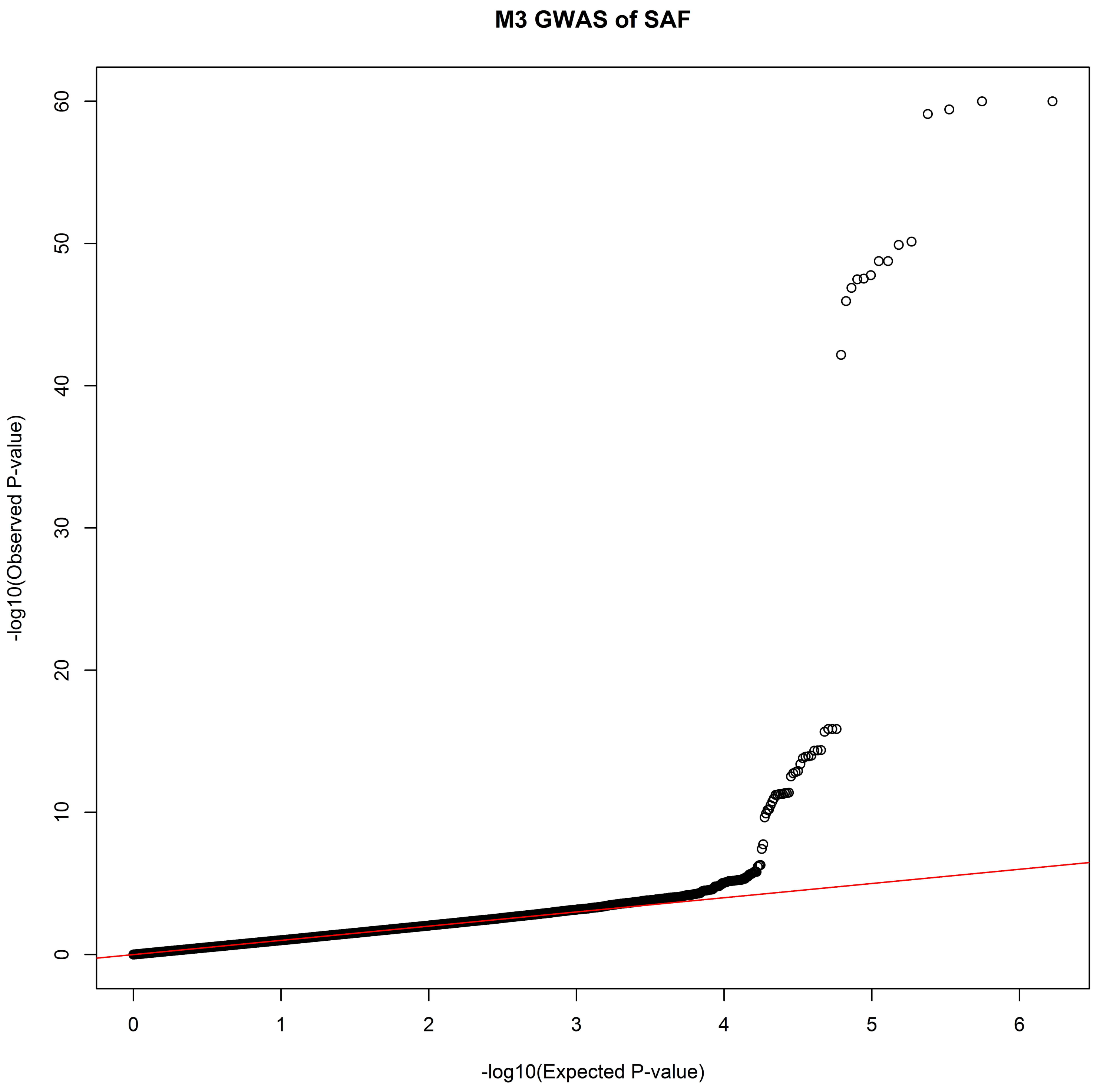
M3 GWAS of SAF
-log10(Observed P-value)
-log10(Expected P-value)
0
10
20
30
40
50
60
0
1
2
3
4
5
6

ESM Figure 4: Q-Q plots of the observed versus expected quantiles of –log10(pvalues) from 837,184 genotyped or imputed (allelic R2≥0.80) SNP associations with SAF in LifeLines adjusted for covariates in M3. Genomic control lambda for SAF was equal to 1.01[1].

[1] Devlin B, Roeder K (1999) Genomic control for association studies. Biometrics 55: 997-1004
