# Supplementary material for: GWAS identifies an NAT2 acetylator status tag single nucleotide polymorphism to be a major locus for skin fluorescence
Source: Diabetologia. 2014 Jun 17;57(8):1623–34. doi: 10.1007/s00125-014-3286-9 (PMC4079945; doi:10.1007/s00125-014-3286-9)
Supplement: Supplementary file 16 — (PDF 175 kb) [file 125_2014_3286_MOESM16_ESM.pdf]

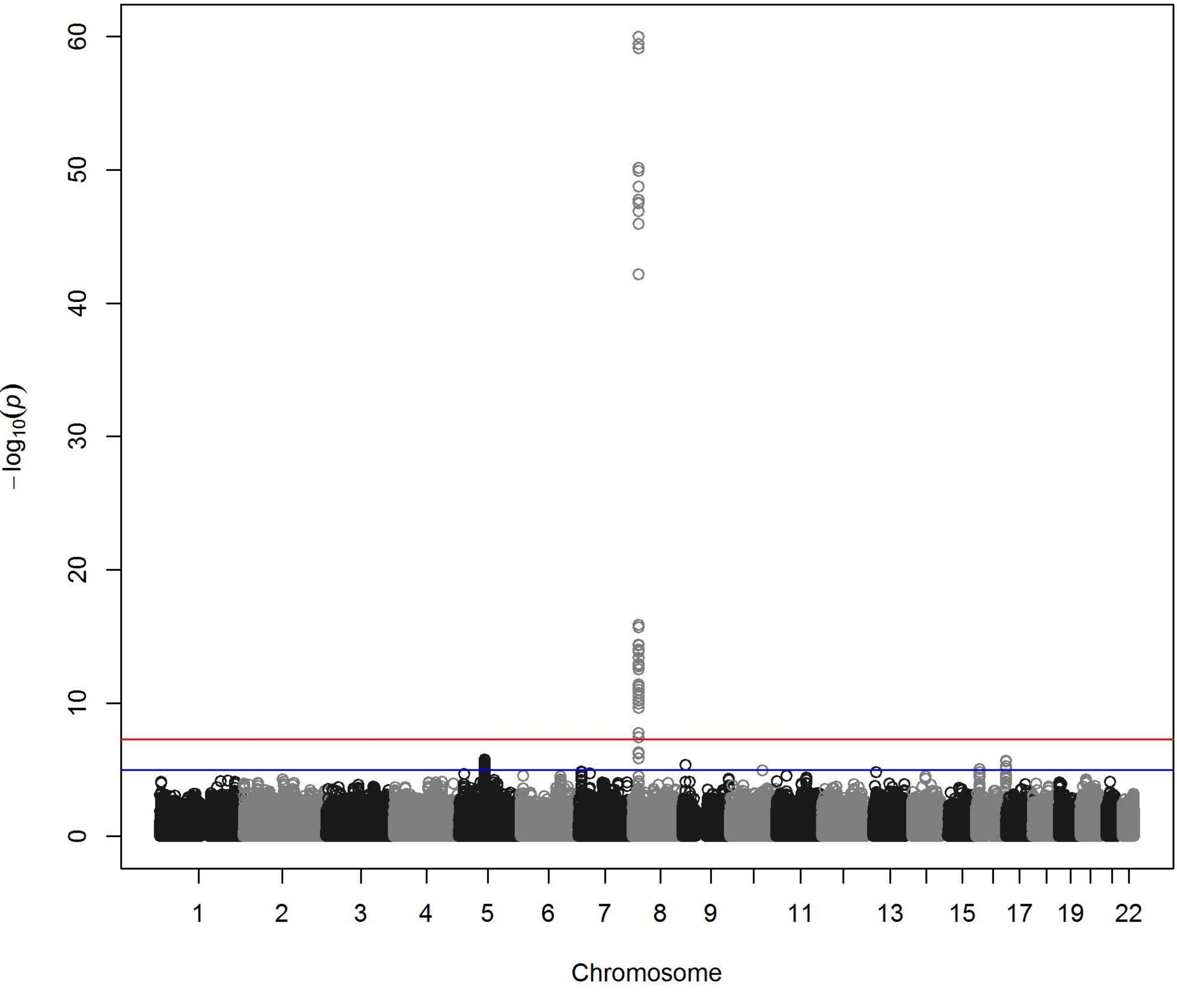

60
50
40
30
20
10
0
$-\log_{10}(p)$
1
2
3
4
5
6
7
8
9
11
13
15
17
19
22
Chromosome

ESM Figure 5: The Manhattan plot for the SAF (M3) GWAS in LifeLines shows $-\log_{10} P$ values (*y*-axis) of 837,184 genotyped or imputed (allelic R2≥0.80) SNPs plotted against their chromosomal positions (*x*-axis). The red horizontal line represents the genome-wide significance threshold, p=5x10-8; the blue line represents p=1x10-5. The Manhattan plot was generated using R code written by Getting Genetics Done (http://gettinggeneticsdone.blogspot.ca/2010/01/gwas-manhattan-plots-and-qq-plots-using.html).
