# Supplementary material for: GWAS identifies an NAT2 acetylator status tag single nucleotide polymorphism to be a major locus for skin fluorescence
Source: Diabetologia. 2014 Jun 17;57(8):1623–34. doi: 10.1007/s00125-014-3286-9 (PMC4079945; doi:10.1007/s00125-014-3286-9)
Supplement: Supplementary file 17 — (PDF 120 kb) [file 125_2014_3286_MOESM17_ESM.pdf]

a.

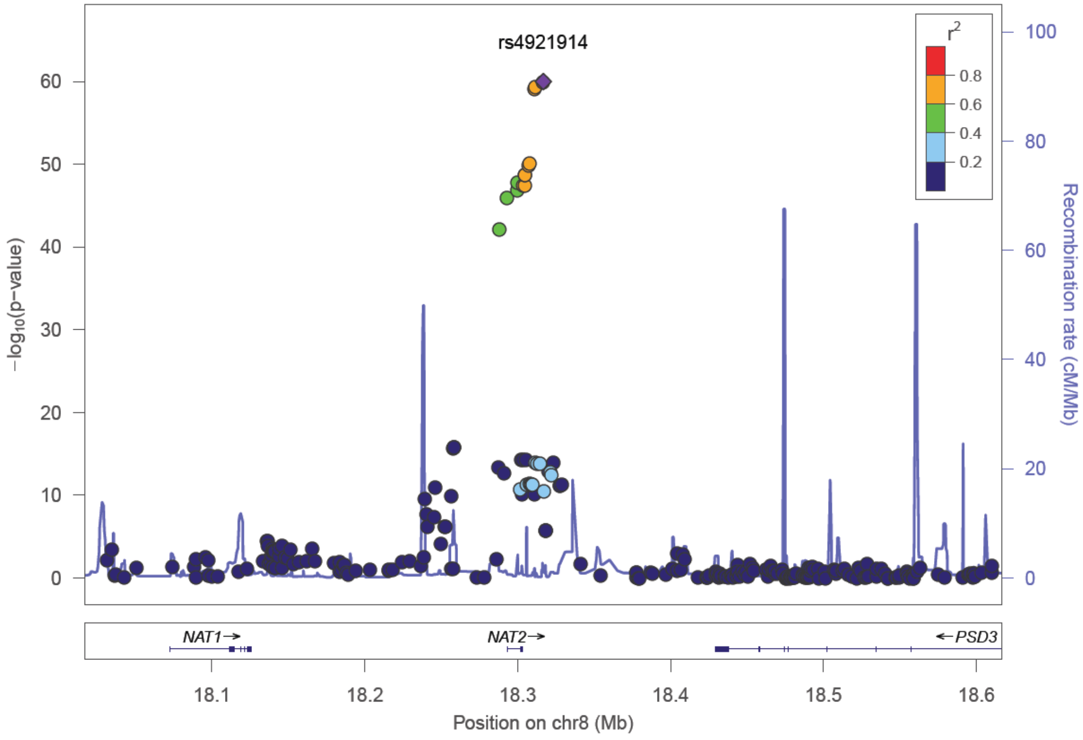
rs4921914
−log10(p−value)
Recombination rate (cM/Mb)
r2
0.8
0.6
0.4
0.2
NAT1→
NAT2→
←PSD3
18.1
18.2
18.3
18.4
18.5
18.6
Position on chr8 (Mb)

b.

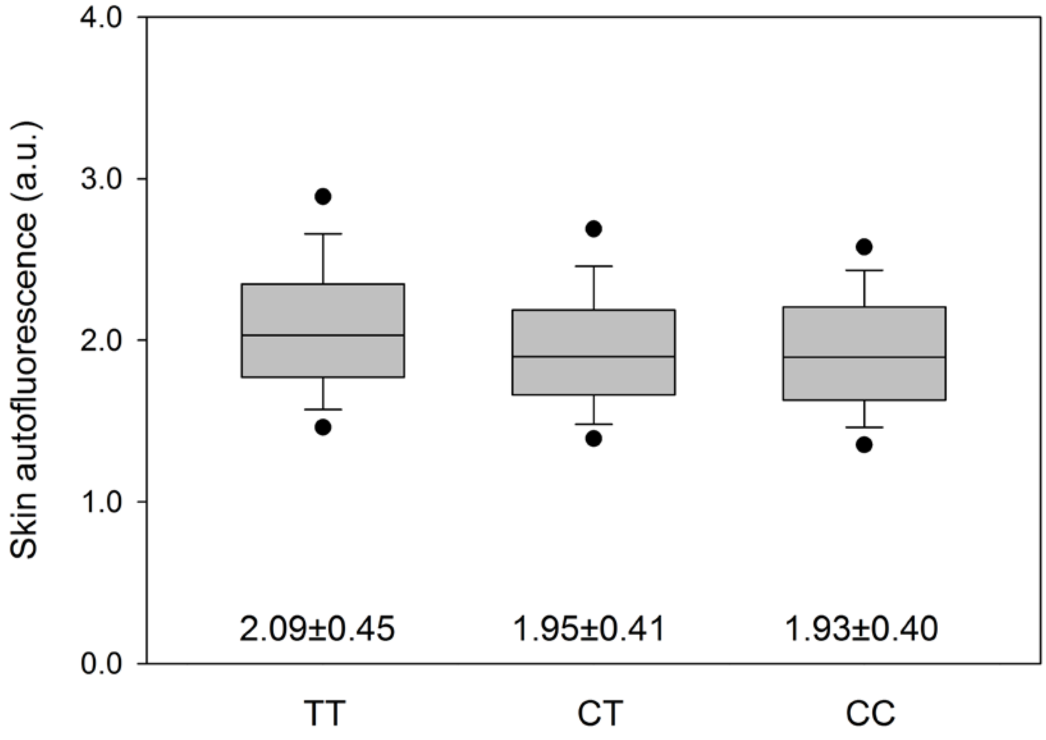
Skin autofluorescence (a.u.)
4.0
3.0
2.0
1.0
0.0
2.09±0.45
1.95±0.41
1.93±0.40
TT
CT
CC

ESM Figure 6: A, Regional plot of a 300-kb region surrounding rs4921914 showing SNPs plotted with their (-log_{10}) p-values from the LifeLines Cohort Study (M3) on the left y-axis and genomic position on the x-axis. Gene annotations are shown above the x-axis and estimated recombination rates (HapMap II release 22) are plotted on the right y-axis. LD values shown were calculated based on pairwise r^2 values for rs4921914 from HapMap CEU phase II (release 22). The blue diamond indicates, rs4921914, and SNPs are colored based on their LD with it. The region plot was generated using LocusZoom (http://csg.sph.umich.edu/locuszoom); **B,** Box plot showing the relationship between imputed rs4921914 genotype and skin autofluorescence in the LifeLines Cohort Study. The box plots depict median and interquartile range, whiskers indicate 10th and 90th percentile. Dots refer to 5th / 95th percentile. Values (mean ± SD of SAF) are given for each genotype group (n=5699 TT/n=2718 CT/n=304 CC) from analysis of variance, F=69.03, p= 2.73 x 10^{-30}).
