# Supplementary material for: GWAS identifies an NAT2 acetylator status tag single nucleotide polymorphism to be a major locus for skin fluorescence
Source: Diabetologia. 2014 Jun 17;57(8):1623–34. doi: 10.1007/s00125-014-3286-9 (PMC4079945; doi:10.1007/s00125-014-3286-9)
Supplement: Supplementary file 18 — (PDF 861 kb) [file 125_2014_3286_MOESM18_ESM.pdf]

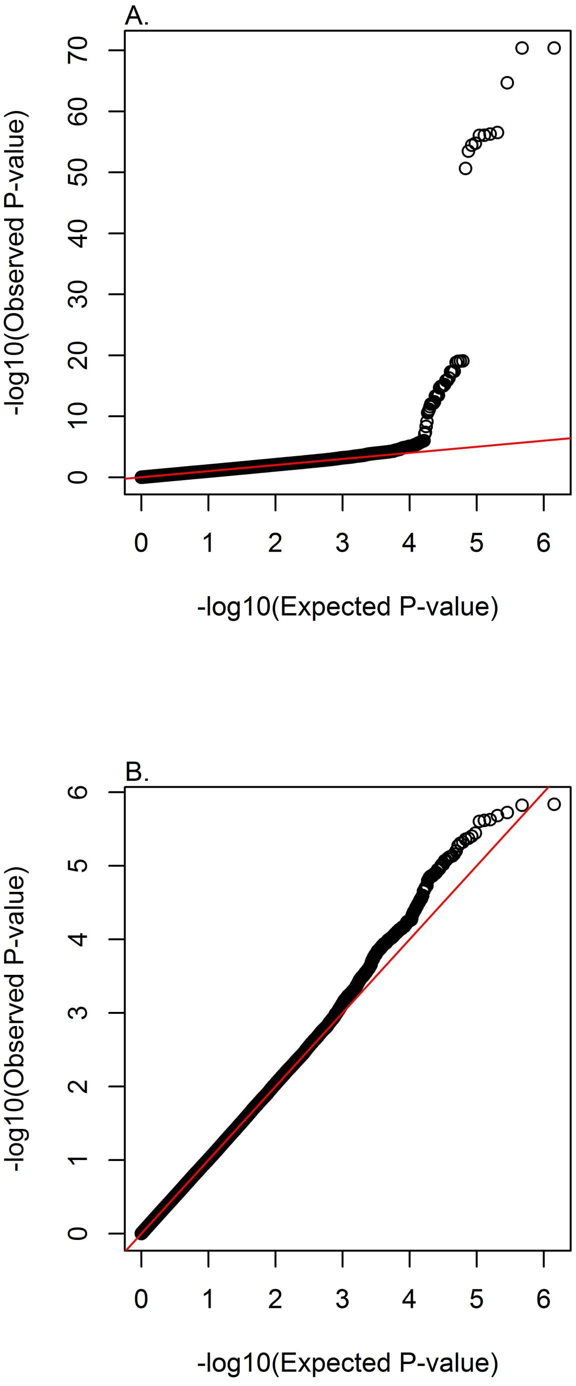
A.
B.
-log10(Observed P-value)
-log10(Expected P-value)
0
10
20
30
40
50
60
70
0
1
2
3
4
5
6

ESM Figure 7: Q-Q plots of the observed versus expected quantiles of –log10(pvalues) from meta-analysis of 715,467 SNPs in DCCT/EDIC and LifeLines analysis of SF (M3). **A,** Plotting all 715,467 SNPs; **B,** Plotting results after excluding 1MB region (874 SNPs) around *NAT2*. Genomic control lambda for both A and B were 1.02 [1].

[1] Devlin B, Roeder K (1999) Genomic control for association studies. Biometrics 55: 997-1004
